# Local genetic correlations between systemic sclerosis and common cancer types

**DOI:** 10.1371/journal.pone.0350006

**Published:** 2026-05-27

**Authors:** Karina Patasova, Weng Ian Che, Helga Westerlind, Lina Marcela Diaz-Gallo, Marie Holmqvist

**Affiliations:** 1 Center for Molecular Medicine, Department of Medicine Solna, Karolinska Institutet, Stockholm, Sweden; 2 Translational and Clinical Research Institute, Newcastle University, Newcastle upon Tyne, United Kingdom; 3 Population Health Sciences Institute, Newcastle University, Newcastle upon Tyne, United Kingdom; 4 Department of Public Health and Medicinal Administration, Faculty of Health Sciences, University of Macau, Macau SAR, China; 5 Division of Clinical Epidemiology, Department of Medicine Solna, Karolinska Institutet, Stockholm, Sweden; 6 Division of Rheumatology, Department of Medicine Solna, Karolinska Institutet and Karolinska University Hospital, Stockholm, Sweden; 7 Medical unit Gastro, Dermatology, Rheumatology, Karolinska University Hospital, Stockholm, Sweden; Nelson Mandela African Institute of Science and Technology, TANZANIA, UNITED REPUBLIC OF

## Abstract

**Introduction:**

The incidence of cancer, encompassing all major types, is significantly higher among patients with systemic sclerosis (SSc) compared to the general population. While previous studies have assessed the global genetic relationships between SSc and various cancers and found no evidence of causality or pleiotropy, these methods average effects across the genome and thus ignore potential opposing directional effects at different loci, thereby missing locus-specific associations. To address this gap, we assessed fine-scaled genetic overlap between SSc and commonly associated cancers, namely breast and lung cancers, as well as hematologic malignancies, by applying local genetic correlation analyses.

**Methods:**

We assessed the genetic relationship between SSc and cancers that frequently co-occur with SSc: breast cancer and its’ four main molecular subtypes: HER2-enriched-like, luminal A and B-like, and triple-negative breast cancers, as well as lung cancer, lymphocytic leukemia, and non-Hodgkin's lymphoma. Published genome-wide association study statistics were obtained from the GWAS catalog and The Breast Cancer Association Consortium, and data were standardized, quality control filtered, and preprocessed. Global and local genetic correlations were evaluated using linkage disequilibrium score regression and Local Analysis of [co]Variant Annotation software. Gene-based cross-trait meta-analysis, colocalization and fine-mapping approaches were used to assess evidence of pleiotropy across regions identified by local genetic correlation analyses. We implemented visualization of the local genetic correlations and functional enrichment analyses of pleiotropic genes from these local correlations.

**Results:**

We did not detect any significant global genetic correlation between SSc and the analyzed cancer subtypes. However, we identified 23 significant local bivariate correlations; 21 were with different molecular subtypes of breast cancer. However, only the locus shared between SSc and lung cancer showed strong evidence of pleiotropy. Genes within loci shared with lung cancer were involved in cell communication and signaling, extracellular matrix remodelling and skin morphogenesis.

**Conclusions:**

We report a pleiotropic locus between SSc and lung cancer, illuminating potential pathobiological mechanisms and providing gene candidates for future research.

## Introduction

Systemic sclerosis (SSc) is an immune-mediated disease primarily affecting women. It is characterized by vasculopathy and abnormally high extracellular matrix deposition in skin and other organs, leading to fibrosis [1,2]. Similar to other immune-mediated disorders, patients with SSc are at greater risk of developing cancer compared to the general population. In patients with SSc, cancer remains the leading cause of death unrelated to SSc, accounting for over a third of fatalities [3]. The types of cancer that are more common in SSc than in the general population differ across populations. Still, the risk of lung cancer, breast cancer, and hematological malignancies is consistently reported as elevated in SSc [4].

Additionally, evidence points to an intriguing temporal relationship between the onset of SSc and cancer development. In particular, there seems to be a biphasic incidence of cancer; the first peak of cancer coincides with SSc diagnosis, occurring within five years before or after SSc onset and the second more than ten years later. Interestingly, breast carcinomas tend to develop during the first peak, while gastrointestinal tract cancers predominate at the second peak [5]. The first cancer incidence peak coinciding with SSc diagnosis suggests that some cancer types are part of a paraneoplastic phenomenon, whereby autoimmunity is triggered by a protective antitumor response against malignant cells in a genetically susceptible host [6–8]. Cancer risk also appears to be mediated by SSc-associated autoantibodies, such as anti-RNA polymerase III (anti-POL3) and anti-topoisomerase (anti-Scl-70), which have been associated with increased cancer risk in these patients [9,10].

The temporal clustering and SSc autoantibodies linked to cancer indicate that there are shared and diverse molecular mechanisms involved in SSc-related cancer. However, our understanding of the molecular mechanisms underlying the pathological link is limited. The few Mendelian Randomization (MR) studies systematically examining the genetic overlap between SSc and cancer, have not found evidence supporting causal or pleiotropic associations. For example, Peng et *al.* reported no causal relationship between SSc and lung cancer [11]. Similarly, Xu and colleagues found no causality between SSc and various breast cancer subtypes in European and East-Asian cohorts [12]. MR analyses examining whether genetically predicted predisposition to immune-mediated diseases could predict risk of non-Hodgkin's lymphoma, did not support causal or pleiotropic associations with SSc [13]. Additionally, one of the few MR studies on SSc and cancer [13], purposefully excluded the Major Histocompatibility Complex (MHC) region due to its complexity. The MHC region, spanning ~3.5–5 Mb on chromosome 6, contains numerous immune-related genes, such as human leukocyte antigen (HLA) alleles, which are involved in both autoimmunity [14] and cancer [15]. By ignoring this region, these studies may miss key pleiotropic signals and biologically pertinent genetic mechanisms shared between SSc and cancer. Overall, MR and genetic correlation methods estimate average effects across the entire genome, which can obscure important signals by averaging out locus-specific associations and masking opposing directional effects at different loci [16], Therefore, methods that assess genetic relationships at a global scale are unable to identify specific regions that may drive associations between SSc and various cancers. To address this gap, our study applied local genetic correlation analyses, examining the genetic overlap of specific loci between SSc and the most common concomitant cancer subtypes, namely breast and lung cancers and hematological malignancies, including non-Hodgkin's lymphoma and lymphocytic leukemia. By focusing on fine-scale genetic overlap, we sought to identify genomic regions of shared susceptibility and shed light on biological pathways shared between SSc and cancer.

## Materials and methods

### Data pre-processing

Genetic markers associated with SSc were obtained from the largest SSc meta-GWAS [17], which comprised 9,846 patients with SSc and 18,333 healthy controls from 14 cohorts, primarily from Western and Northern European countries and the USA. Full details are outlined elsewhere [17], but in brief, cases were required to fulfill the 1980 American College of Rheumatology [18] or LeRoy and Medsger criteria [19]. Different versions of Illumina and Affymetrix arrays were used for genotyping, and following quality-control procedures, 4,723,365 SNPs were analyzed [17].

We included genetic data on breast [20] and lung cancers [21], lymphocytic leukemia, and non-Hodgkin's lymphoma [22]. Given the high heterogeneity of breast tumors [23], which significantly impacts cancer behavior, we investigate the genetic correlations between SSc and four main molecular subtypes of breast cancer, HER2-enriched-like, luminal A and B-like, including HER2-negative subtype, and triple-negative breast cancers.

These GWAS were focused on individuals of European descent and were obtained from the GWAS catalog [24] and Breast Cancer Association Consortium [25]. GWAS on breast, lung cancers, and lymphocytic leukemia/non-Hodgkin's lymphoma were respectively performed using OncoArray [20], custom genome-wide Illumina [21], and different versions of Axiom GWAS arrays [22].

Data standardization and quality control were performed using Bioconductor *MungeSumstats* R package [26] in R version 4.3.2 [27]. Specifically, summary statistics were aligned to the reference genome (1000 genomes Phase2 Reference Genome Sequence hs37d55; ‘*BSgenome.Hsapiens.1000genomes.hs37d5*’ R package [28]), and alleles were flipped to match the reference file (‘*SNPlocs.Hsapiens.dbSNP155.GRCh37’* R package [29]). SNPs absent from the reference file and variants with betas and p-values equal to zero were excluded. Where necessary, standard errors were calculated from betas and p-values using *impute_se* command. The pipeline of the study analyses is shown in S1 Fig. Table 1 presents information on SSc and cancer GWAS summary statistics after standardization and quality control steps.

**Table 1:** Information about GWAS summary statistics utilized in genetic correlation analyses.

Traits (Reference)	Total sample size	N cases	N controls	N markers in GWAS	N markers after QC
Systemic sclerosis [17]	26,679	9,095	17,584	4,723,365	4,645,601
Breast cancer [20]	247,173	133,384	113,789	10,760,768	9,299,447
HER2-enriched-like breast cancer [20]	175,475	69,501	105,974	11,792,543	10,130,149
Luminal A-like breast cancer [20]	136,73	45,253	91,477	9,965,311	8,476,164
Luminal B-like breast cancer [20]	97,904	6,427	91,477	9,965,311	8,476,164
Luminal B HER2-negative-like breast cancer [20]	97,827	6,350	91,477	9,965,311	8,476,164
Triple-negative breast cancer [20]	100,079	8,602	91477	9,965,311	8,476,164
Lung cancer [21]	85,716	29,266	56,45	10,439,017	7,661,078
Lymphocytic leukemia [22]	411,202	852	410,350	9,987,596	8,860,344
Non-Hodgkin's lymphoma [22]	412,750	2,400	410,350	9,987,596	8,860,344

The column “Traits (Reference)” lists the names of analyzed traits with a study reference. The total number of genome-wide association study (GWAS) participants, cases and controls is displayed in the fields “Total sample size,” “N cases,” and “N controls,” respectively. “N markers in GWAS” and “N markers after QC” are the number of genetic variants in GWAS summary statistics and after quality control (QC), respectively.

## Statistical analyses

### Global genetic correlations

To calculate the total genetic correlation between SSc and breast cancer, lung cancer, and hematological malignancies, we used an approximation method implemented in the linkage disequilibrium score regression (LDSC) software where LD scores are generated from the 1000 Genomes Project Phase 3 European population panel [30–32]. In LDSC analyses, summary statistics from GWAS were additionally processed with HapMap Project Phase 3 SNPs [33] using *munge_sumstats.py* script [34] Specifically, variants with MAF < 1% and SNPs within the extended MHC locus (chromosome 6: 20,000,000–40,000,000) were excluded. Genetic correlations with p-value < 0.05 were considered statistically significant.

### Local genetic correlations

Specific loci contribution was assessed through pairwise local genetic correlations across numerous loci, using the Local Analysis of [co]Variant Annotation (LAVA) software [16]. LAVA was included as one of the main methods in our study due to its robust capabilities in assessing local genetic correlations. Traditional genetic correlation analyses methods such as LDSC regression often focus on global correlations, which can obscure important local variations. LAVA addresses this limitation by analyzing genetic correlations at specific genomic loci, providing a more comprehensive understanding of the genetic architecture underlying complex traits and providing gene candidates for future functional studies [16]. Moreover, the complex MHC region, which is very important for autoimmune diseases, can be included in LAVA analyses, whereas in global correlation analyses, it is often excluded. LAVA analyses were performed on 2,495 loci, which were generated by Werme *et al.* by partitioning the whole genome into equal-sized semi-independent LD blocks with at least 2,500 SNPs and LD between SNPs less than 0.25 [16]. To control for potential sample overlap between GWASs, LAVA analyses included a sample overlap file generated using LDSC [30,31].

To identify relevant loci, associations between individual diseases and 2,495 loci, including the MHC region, were evaluated using a univariate genetic correlation test and loci that showed evidence of significant local heritability (p-value < 2.0 x10^-05^ (0.05/2,495)) for both traits in the SSc-cancer pairs were advanced for bivariate local genetic correlation analyses. Local bivariate genetic correlations were considered statistically significant if their Bonferroni-adjusted p-values were < 0.05. Bonferroni-adjusted p-values were calculated using *p.adjust* function in R [27] based on the number of loci that passed univariate tests performed for each SSc-cancer pair.

Following the assessment of bivariate local genetic correlations, regional SNPs-level associations within loci shared between SSc and cancers were visualized using *topr* package [35] in R [27]. The replication of significant bivariate genetic correlations was performed using publicly available SSc GWAS summary statistics published by FinnGen [36]. Similarly, Bonferroni-adjusted p-values were used to determine the significance of local genetic correlations.

### Gene-based pleiotropy cross-trait meta-analysis

To investigate gene-level evidence of pleiotropy across regions shared with cancer, we performed cross-trait gene-set association analysis using *GCPBayes* [37,38]. *GCPBayes* aggregated SNP-level association signals into gene-level Bayes factors and estimated posterior probabilities of a gene (group) or SNPs assigned to a gene (within group) being truly associated with both traits. Hierarchical Bayesian model with hierarchical spike (HS) and slab priors facilitated variable selection by distinguishing relevant from irrelevant SNPs while accounting for LD and gene size [37,38]. To assess cross-trait associations at a gene/group level, SNPs were annotated to genes using protein-coding gene coordinates. LD clumping was performed to mitigate redundancy in LD blocks [37,38]. The clumping procedure was performed on SNPs with p-value ≤ 0.99 using r² threshold of 0.6 and a 10 Mb window to identify independent SNPs. Genes were considered strongly associated with both traits if they satisfied criteria of log_10_Bayes Factor (BF) > 1, Local Bayesian False Discovery Rate (lBFDR) < 0.05 and met pleiotropy criteria derived from both the posterior median and the credible interval. Significant results produced by the analyses using the Dirac spike function were confirmed by running Gibbs sampler with HS.

### Colocalization

We performed Bayesian colocalization analyses to determine whether genetic signals at pleiotropic loci were driven by a common causal variant shared between SSc and each cancer type. Bayesian colocalization analyses, performed in *coloc* R package [39], calculated posterior probabilities for 5 different hypotheses [40]:

H0: No association with either trait;

H1: Association with SSc only;

H2: Association with cancer only:

H3: Association with both traits, but different causal variants:

H4: Association with both traits, same causal variant (colocalization).

Posterior probabilities (PP.H0–PP.H4) were calculated for each pair of traits and regions, separately, with PP.H4 ≥ 0.8 indicating the presence of a shared causal SNP, whereas high PP.H3 favoured pleiotropy without shared causality.

### Fine mapping

We applied SuSiE (Sum of Single Effects) regression for fine-mapping within regions showing evidence of pleiotropy [40]. SuSiE identified credible sets of variants likely to contain causal SNPs by modelling multiple effects simultaneously [40]. For each region, the top hits and their posterior inclusion probabilities (PIPs) were reported [40].

### Functional exploration of significant bivariate genomic correlations

We investigated possible functional implications of pleiotropic genes identified in *GCPBaye*s analyses in functional enrichment analyses using *gprofiler2* R package [41]. The *gprofiler2* R package employed data from various sources, including Gene Ontology (GO) [42] and Kyoto Encyclopedia of Genes and Genomes (KEGG) [43]. Biological pathways and functions with FDR p-value < 0.05 were considered statistically significant. Results of functional analyses were visualized using *clusterProfiler* [44] and *enrichplot* [45] R packages*.*

## Results

### Global genetic correlations

Global genetic correlations between SSc and breast and lung cancers, lymphocytic leukemia and non-Hodgkin's lymphoma, including different molecular subtypes, were assessed in LDSC-regression. We did not find evidence supporting global genetic correlations between SSc and the studied cancer subtypes (Table 2).

**Table 2:** Global genetic correlations between SSc and different cancer subtypes.

Trait paired with SSc	Genetic correlation coefficient	se	p-value
Breast cancer	0.06	0.05	0.3
HER2-enriched-like breast cancer	0.01	0.05	0.8
Luminal A-like breast cancer	0.01	0.05	0.8
Luminal B-like breast cancer	0.03	0.05	0.6
Luminal B HER2-negative-like breast cancer	0.01	0.05	0.8
Triple-negative breast cancer	0.07	0.05	0.2
Lung cancer	0.02	0.07	0.8
Lung adenocarcinoma	−0.12	0.09	0.2
Non-Hodgkin's lymphoma	0.12	0.15	0.4
Lymphocytic leukemia	0.47	0.28	0.1

The column “Trait paired with SSc” lists traits analyzed with SSc. The columns “Genetic correlation coefficient,” “se” and “p-value” include the genetic correlation coefficient, standard error, and probabilities.

### Local genetic correlations

**Fig 1:**
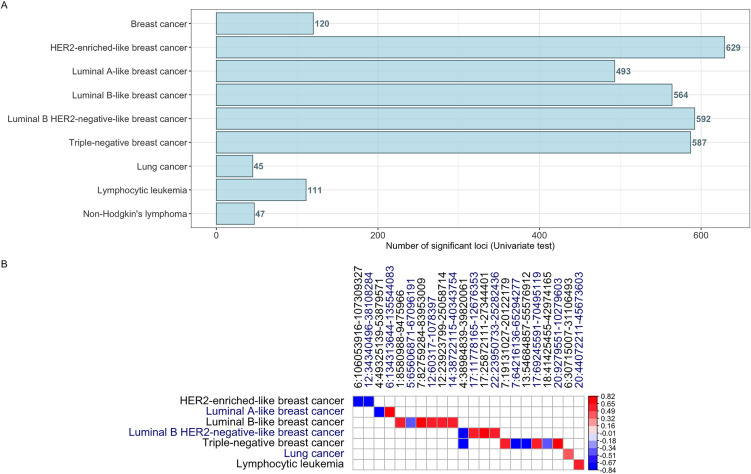
Results of univariate and bivariate LAVA analyses. Panel A displays the number of loci that passed univariate test and showed significant local heritability for both SSc and the studied cancers. Panel B shows Bonferroni-significant local bivariate genetic correlations.

LAVA analyses were applied to breast cancer and its molecular subtypes, lung cancer, lymphocytic leukemia, and non-Hodgkin’s lymphoma. Fig 1A displays the number of loci that displayed significant local heritability for both SSc and each studied cancer.

We detected twenty-three significant local genetic correlations passing the threshold for statistical significance (Table 3; Fig 1B); 21 of them were with different molecular subtypes of breast cancer, including HER2-enriched-like, luminal A-like, luminal B-like, luminal B HER2-negative-like, and triple-negative breast cancers. Specifically, SSc displayed strong negative genetic correlations with HER2-enriched-like breast cancer across two loci on chromosome 6 and 12, respectively (chr6:106053916–107309327, genetic correlation coefficient (r_g_)=−0.8; chr12:34340496–38108284, r_g_ = −0.8). Similarly, SSc and luminal A-like breast cancer also shared a locus on chromosome 6 (chr6:134313644–135544083) with a positive genetic correlation of 0.8 and a locus on chromosome 4 that showed a negative genetic correlation of −0.8. Positive local genetic correlations ranging from 0.5–0.8 were detected between SSc and luminal B-like breast cancer across five loci on chromosomes 1, 7, 12, and 14. In addition to these, another common locus was mapped to chromosome 5 (chr5:65606871–67096191), exhibiting a negative genetic correlation of −0.5. There were four loci shared between SSc and luminal B HER2-negative-like breast cancer, of which only one displayed a negative genetic correlation (chr4: 38984839−39820061; r_g_ = −0.8), and the remaining three local genetic correlations on chromosomes 17 and 22 varied between 0.6 and 0.8, respectively. We also identified three positive genetic correlations between SSc and triple-negative breast cancer across three distinct loci on chromosomes 7, 17, and 20; these genetic correlations ranged between 0.6–0.8. Four negative genetic correlations were also observed across loci on chromosomes 4, 7, 13, and 18, ranging between −0.8 and −0.4. In contrast to numerous common loci identified between SSc and breast cancer, lung cancer and lymphocytic leukemia each shared a single locus with SSc mapped to chromosomes 6 and 20, accordingly (r_g_ = 0.4 for lung cancer and r_g_ = 0.6 for lymphocytic leukemia). We did not detect significant local genetic correlations between SSc and non-Hodgkin's lymphoma.

**Table 3:** Significant local genetic correlations between SSc and different cancers.

Trait paired with SSc	loci	N SNPs	Genetic correlation coefficient (95% CI)	r2 (95% CI)	p-value	Bonferroni-adjusted p-value
HER2-enriched-like breast cancer	6:106053916-107309327	2064	−0.8 (−1,-0.5)	0.6 (0.3,1)	1.6x10^-07^	0.0001
	12:34340496-38108284	1533	−0.8 (−1,-0.5)	0.6 (0.3,1)	1.8x10^-05^	0.01
Luminal A-like breast cancer	4:49325139-53879571	1298	−0.8 (−1,-0.5)	0.7 (0.3,1)	1.8x10^-05^	0.009
	6:134313644-135544083	1313	0.8 (0.5,1)	0.6 (0.2,1)	8.8x10^-06^	0.004
	1:8580988-9475966	718	0.6 (0.4,0.8)	0.3 (0.1,0.6)	1.0x10^-06^	0.001
Luminal B-like breast cancer	5:65606871-67096191	2333	−0.5 (−0.7,-0.3)	0.2 (0.1,0.6)	2.8x10^-05^	0.02
	7:82759284-83953009	2365	0.8 (0.6,1)	0.7 (0.3,1)	2.3x10^-07^	0.000
	12:60317-1078397	955	0.6 (0.3,0.8)	0.3 (0.1,0.7)	5.7x10^-05^	0.03
	12:23923799-25058714	1740	0.7 (0.4,1)	0.4 (0.2,0.9)	7.2x10^-06^	0.004
	14:38722115-40343754	2848	0.5 (0.3,0.8)	0.3 (0.1,0.6)	3.7x10^-06^	0.002
Luminal B HER2-negative-like breast cancer	4:38984839-39820061	1211	−0.8 (−1,-0.5)	0.6 (0.3,1)	1.3x10^-05^	0.008
	17:11778165-12676353	877	0.6 (0.3,0.8)	0.3 (0.1,0.7)	2.5x10^-05^	0.01
	17:25872111-27344401	989	0.8 (0.5,1)	0.7 (0.3,1)	6.2ex10^−06^	0.004
	22:23950733-25282436	1558	0.6 (0.4,0.9)	0.4 (0.1,0.8)	7.8x10^-06^	0.005
Triple-negative breast cancer	4:38984839-39820061	1211	−0.7 (−1,-0.4)	0.5 (0.2,1)	6.3x10^-05^	0.04
	7:19131027-20122179	2084	0.7 (0.4,1)	0.4 (0.2,0.9)	9.4x10^-06^	0.006
	7:64216136-65294277	1446	−0.8 (−1,-0.6)	0.7 (0.3,1)	5x10^-06^	0.003
	13:54684857-55576912	1499	−0.7 (−1,-0.5)	0.6 (0.2,1)	7.5x10^-06^	0.004
	17:69245591-70495119	1440	0.6 (0.3,0.8)	0.3 (0.1,0.7)	8.3x10^-06^	0.005
	18:41425455-42974165	2060	−0.4 (−0.6,-0.2)	0.2 (0.1,0.4)	1.6x10^-05^	0.009
	20:9279551-10279603	1472	0.8 (0.4,1)	0.6 (0.2,1)	5.1x10^-05^	0.03
Lung cancer	6:30715007-31106493	2005	0.4 (0.2,0.6)	0.2 (0,0.4)	5.1x10^-04^	0.02
Lymphocytic leukaemia	20:44072211-45673603	2232	0.6 (0.4,0.9)	0.4 (0.1,0.8)	1.4x10^-05^	0.002

Field “Trait paired with SSc” lists cancers analyzed in bivariate tests. The genomic positions of analyzed loci are shown as chromosome numbers, as well as start and end base pair positions. The number of total SNPs within a locus is shown in field “N SNPs.” Standardized coefficients of the local genetic correlations, their 95% confidence intervals and p-values are respectively displayed in columns “Genetic correlation coefficient (95% CI)” and “p-value.” The field “r2 (95% CI)” represents the proportion of variance in the genetic signal for cancer (the “Trait paired with SSc”) that is explained by systemic sclerosis, with the corresponding 95% confidence interval also provided. Column “Bonferroni-adjusted p-value” includes p-value after Bonferroni adjustment.

**Fig 2:**
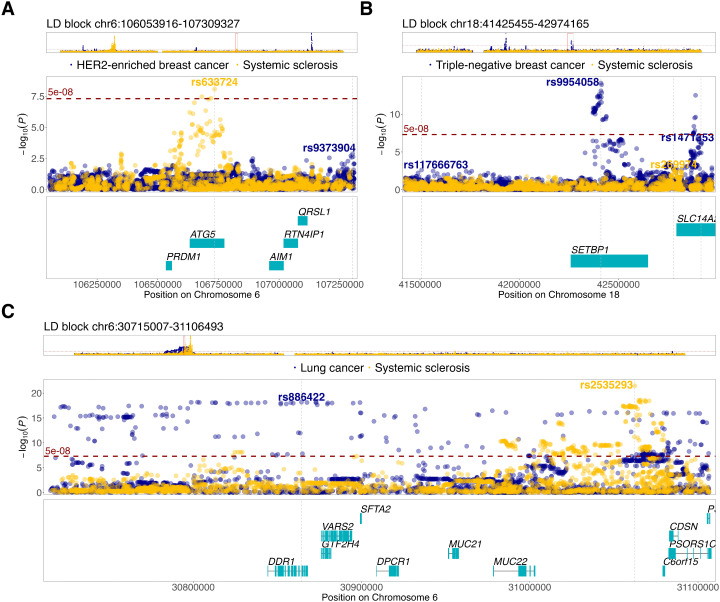
Regional plots of loci that showed significant local genetic correlations in LAVA analyses and contained genome-wide significant SNPs specific to SSc and the studied cancer types. Genes were mapped to the loci shared between SSc and breast cancer subtypes (panels A & B) and lung cancer (panel **C)**. Strongest genetic associations within the loci are marked with SNP rsid names. Genetic association probabilities are plotted as –log_10_ (p-value) on the y-axis against chromosomes on x-axis.

Most of these loci did not contain SNPs associated at genome-wide significance threshold (p < 5 x 10^-08^) for SSc or breast cancer. For instance, genomic locus chr6:106053916–107309327 carried genetic markers associated with SSc at genome-wide significance but this was not the case for HER2 enriched like breast cancer (Fig 2A). By contrast, the locus chr18:41425455–42974165 harbored SNPs that showed genome wide-significant SNP associations with triple-negative breast cancers but not SSc (Fig 2B). The locus chr6:30715007–31106493 carried SNPs associated at genome-wide significance level with SSc and lung cancer, mirrored by local genetic correlation between these two traits (Table 3, Fig 2C).

### Gene-based cross-trait meta-analysis

Of the 21 genomic regions flagged by LAVA genetic correlation analyses, GCPBayes found evidence of gene-level pleiotropy in two. In the locus shared with the lung cancer (6:30715007–31106493), ten genes, (*PSORS1C2*, *C6orf15*, *LINC02570, DDR1-DT*, *MUC21*, *CDSN*, *DDR1*, *PSORS1C1, HCG20*, and *MUC22),* were classified as pleiotropic under GCPBayes confidence interval and median-based criteria and confirmed by HS-based analyses (S2 Table). In contrast, the lymphocytic leukaemia locus (20:44072211–45673603) exhibited a more focused association signal, with only one gene, *SLC12A5*, implicated based on median criteria. Theta (0.6) for this gene indicated a moderate shared signal (S1 Table). Loci shared with breast cancers, such as HER2-enriched-like, Luminal A-like, Luminal B-like, Luminal B HER2-negative-like, and triple-negative subtypes lacked detectable pleiotropic effects under the applied *GCPBayes* framework, with significant regional associations driven mostly breast cancer-specific signals (PPA phenotype > 0.5) (S2 Table)

### Colocalization

In the 6p locus (6:30715007–31106493), identified by *GCPBayes* analyses as containing pleiotropic genes, colocalization analyses strongly supported distinct causal variants associated with SSc and lung cancer (PP.H3 ≈ 1.0), while the probability of a shared causal variant (PP.H4) was zero (S2 Fig, Panel A)

### Fine-mapping

Across 6p locus (6:30715007–31106493), fine-mapping identified multiple SNPs with PP.H3 = 1.0 (e.g., rs886422 and rs9263631). Given all of these SNPs had PP.H4 close to 0, SuSiE findings were congruent with coloc analyses which supported strong pleiotropy with distinct causal SNPs for SSc and lung cancer (S2 Fig, Panel B).

### Functional analyses

**Fig 3:**
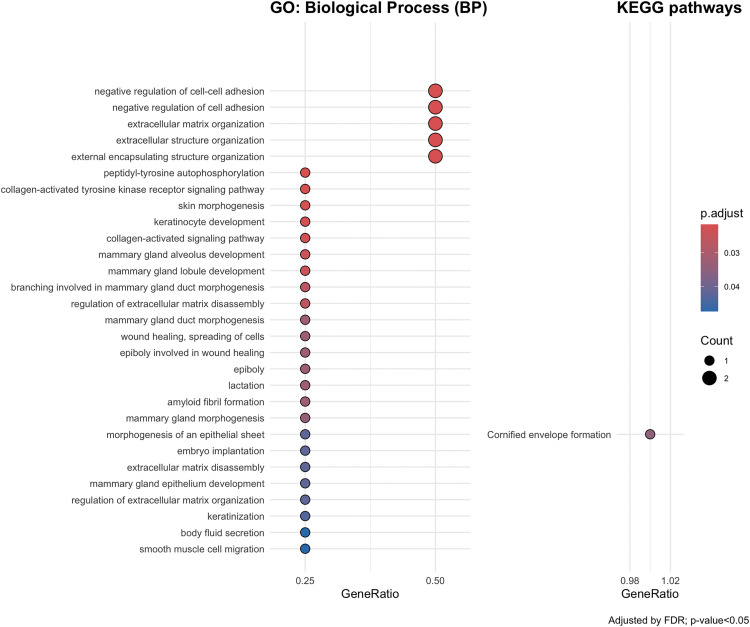
Functions and pathways enriched for pleiotropic genes shared between SSc and lung cancer. Gene-set enrichment analyses used Gene Ontology (GO) and Kyoto Encyclopedia of Genes and Genomes (KEGG) databases.

Gene-set enrichment analyses showed that pleiotropic genes mapped to the 6p locus (6:30715007–31106493), which was shared between SSc and lung cancer, participated in biological processes related to cellular communication, including negative regulation of cell adhesion and extracellular matrix organization among other. Collagen-activated signalling and tyrosine kinase activity pathways were also implicated. Additionally, pleiotropic genes were involved in developmental programs and morphogenesis, as highlighted both by GO and KEGG analyses, as well as wound healing (Fig 3, S3 Table).

### Replication

Replication of significant local genetic correlations included 619 patients with SSc and 365,533 matched controls [36]. None of genetic correlations within twenty-tree analyzed loci reached the Bonferroni significance level (p-value = 0.025) (Table 4).

**Table 4:** Replication of significant local genetic correlations between SSc and different cancers.

Trait paired with SSc from FinnGen (N cases = 680; N controls = 399,355)	loci	N SNPs	Genetic correlation coefficient (95% CI)	r2 (95% CI)	p-value	Bonferroni-adjusted p-value
HER2-positive breast cancer (N cases = 69,501; N controls = 105,974)	6:106053916-107309327	3404	0.1 (−0.1,0.2)	0 (0,0)	0.3	0.3
Luminal A-like breast cancer (N cases = 45,253; N controls = 91,477)	4:49325139-53879571	2046	−0.4 (−0.9,0)	0.2 (0,0.7)	0.03	0.1
	6:134313644-135544083	2662	0.2 (0,0.5)	0 (0,0.2)	0.1	0.2
Luminal B-like breast cancer (N cases = 6,427; N controls = 91,477)	1:8580988-9475966	1924	0 (−0.1,0.2)	0 (0,0)	0.6	>0.999
	5:65606871-67096191	3528	−0.1 (−0.3,0.1)	0 (0,0.1)	0.4	>0.999
	7:82759284-83953009	3196	0.1 (−0.2,0.3)	0 (0,0.1)	0.5	>0.999
	12:60317-1078397	2366	−0.1 (−0.2,0.1)	0 (0,0)	0.4	>0.999
	12:23923799-25058714	2909	0 (−0.3,0.2)	0 (0,0.1)	0.6	>0.999
Luminal B-like HER2-negative breast cancer (N cases = 6,350; N controls = 91,477)	4:38984839-39820061	1880	0 (−0.4,0.5)	0 (0,0.3)	0.8	>0.999
	17:11778165-12676353	1976	0 (−0.1,0.1)	0 (0,0)	0.9	>0.999
	17:25872111-27344401	1935	0.2 (0,0.5)	0.1 (0,0.2)	0.04	0.2
	22:23950733-25282436	2849	0.1 (−0.1,0.2)	0 (0,0.1)	0.3	>0.999
Triple-negative breast cancer (N cases = 8,602; N controls = 91,477)	4:38984839-39820061	1880	0 (−0.5,0.4)	0 (0,0.3)	0.9	>0.999
	7:19131027-20122179	3403	0 (−0.1,0)	0 (0,0)	0.5	>0.999
	13:54684857-55576912	2043	0 (−0.3,0.2)	0 (0,0.1)	0.7	>0.999
	18:41425455-42974165	3066	0 (−0.1,0.1)	0 (0,0)	0.9	>0.999
	20:9279551-10279603	2528	0.2 (0,0.6)	0.1 (0,0.3)	0.1	0.4
Lung cancer (N cases = 29,266; N controls = 56,450)	6:30715007-31106493	2312	−0.2 (−0.6,0.1)	0 (0,0.3)	0.2	0.2
Lymphocytic leukemia (N cases = 850; N controls = 410,350	20:44072211-45673603	3779	0 (−0.1,0.1)	0 (0,0)	0.5	0.5

Field “Trait paired with SSc” lists cancers analyzed in bivariate tests. The genomic positions of analyzed linkage disequilibrium loci are shown as chromosome numbers, as well as start and end base pair positions. The number of total SNPs within a locus is shown in field “N SNPs.” Standardized coefficients of the local genetic correlations and their 95% confidence intervals and p-values are respectively displayed in columns “Genetic correlation coefficient (95% CI)” and “p-value.” The field “r2 (95% CI)” represents the proportion of variance in the genetic signal for cancer (the “Trait paired with SSc”) that is explained by systemic sclerosis, with the corresponding 95% confidence interval also provided. Column “Bonferroni-adjusted p-value” includes p-value after Bonferroni adjustment.

## Discussion

In this study, we sought to examine the genetic relationship between SSc and cancer by applying genetic correlation analyses to GWAS data on SSc and related cancers. We did not find evidence supporting global genetic correlations between SSc and the nine evaluated cancers comprising breast cancer, including HER2-enriched-like, luminal A-like, luminal B-like, luminal B HER2-negative-like and triple-negative molecular groups, lung cancer, lymphocytic leukemia, and non-Hodgkin's lymphoma. Nonetheless, we detected twenty-three significant local genetic correlations between SSc and seven types of cancers; twenty-one were shared with different breast cancer molecular subtypes. Subsequent gene-based cross-trait meta-analysis found sufficient evidence for pleiotropy only at the locus shared with lung cancer, providing gene candidates whose utility can be further explored in functional and clinical studies. Nonetheless, we were not able to replicate observed local genetic correlation using data from FinnGen cohort. Given that FinnGen represents genetically distinct population shaped by founder bottlenecks – combined with a small number of SSc cases in the replication cohort, statistical power was limited in validation analyses. However, currently, no other larger GWAS on SSc are available.

Although published research indicates that SSc and cancer may share several common mechanisms, previous studies found minimal genetic correlations between SSc and different cancer types. Nonetheless, it has been demonstrated that local genetic correlations may be present without significant genome-wide correlation estimates or diverge from global correlations [46], therefore providing a motivation for exploring genetic pleiotropy on the local scale.

In our study, we did not observe any significant global correlations between SSc and the studied cancer types, consistent with previous investigations that used various bioinformatic approaches to assess the genetic relationships. A recently published meta-analysis aggregating SSc GWAS data from European cohorts and Biobank of Japan measured genetic correlations between SSc and 60 different traits, including several cancers [47]. This meta-analysis reported non-significant global correlations for breast cancer (0.17) and hematological malignancies (0.45) [47]. In a MR study, Lu and colleagues [48] examined a causal link between different immune-mediated diseases and breast cancer, finding an OR of 1.00 for SSc. The authors concluded that there was no causal association between SSc and increased risk of developing breast cancer, supporting that some shared environmental and genetic factors could explain previously observed associations [48]. Similarly, MR analyses evaluating the relationship between SSc and lung cancer did not support causality between these traits [11]. Additional stratified analyses did not identify significant associations with lung adenocarcinoma, squamous cell, or small-cell subtypes [11].

Our study identified several loci with significant local genetic correlations between SSc and various subtypes of breast cancer. These local genetic correlations were both positive and negative, suggesting a complex genetic relationship between SSc and cancer. Broadly, positive genetic correlations between SSc and breast cancer at observed loci suggested that genetic variants in a particular region increased the risk for both diseases and contributed to their development. Conversely, negative genetic correlations implied that genetic markers in a particular locus may have opposite effects on SSc and analysed cancers. Negative genetic correlations could hint at different biological mechanisms or pathways influenced by the same genetic region in opposite directions. While numerous loci showed significant local genetic correlations between SSc and various breast cancer subtypes, no consistent evidence of pleiotropy emerged from these local correlations. These discrepancies could be explained by several factors. For instance, trait causality or mediated pleiotropy, where a trait causally influences another trait rather than because genetic variants directly affect both diseases or horizontal pleiotropy [49]. Genetic correlations between traits can arise from pleiotropy or from linkage disequilibrium alone [50]. Additionally, observed negative genetic correlations could indicate antagonistic pleiotropy, in which SNPs within a specific locus increase the risk of one disease while decreasing the risk of another [51]. *GCPBayes* methodology employed Bayesian sparse group sampling that aggregated SNP-level evidence of horizontal pleiotropy at gene level, which assumed co-directionality of the SNP effects [37,38]. Conversely, divergent SNP associations diluted evidence of pleiotropy, resulting in low posterior probabilities of shared effects [37,38]. Both, LAVA or *GCBayes* did not distinguish between vertical and horizontal pleiotropy [16,37,52,53]. LAVA detected regions with genetic correlations, but could not establish whether those arose from pleiotropy or coincidental linkage disequilibrium [16]. This limitation stems from LAVA focus on the statistical associations without a mechanistic insight. The inclusion of expression data could clarify pathways underlying local genetic correlations, providing mechanistic explanations and generating a testable hypothesis for experimental validation.

Overall, both genetic and environmental factors are postulated to influence the association between SSc and breast cancer. Published epidemiological research indicates that breast cancers are typically diagnosed before or shortly after diagnosis of SSc, occurring within a 3–5 years window [5]. Interestingly, mutations in *POLR3A* gene and co-occurring anti-RNA polymerase III autoantibodies were linked to emergence of breast cancer closely to SSc diagnosis [8,54], which supports the role of genetics in paraneoplastic relationship with breast cancer, By contrast, cancer surfacing after diagnosis of SSc would point to potential influence from immunosuppressive medications, such as cyclophosphamide, mofetil and azathioprine, several of which were implicated in cancer [54–58]. Additionally, the temporal interplay between SSc and breast cancer can be a by-product of intense cancer screening of newly diagnosed patients with SSc. It is also important to acknowledge that although genetic correlations may provide novel insights into shared genetics between SSc and cancer, stratifying patients with SSc by their autoantibody profile, comparing the genetic background of patients with SSc that develop malignancies with those that did not and performing functional studies are necessary to validate the biological relevance of these loci. Experimental validation can help confirm whether the identified genetic variants directly influence the risk of SSc-related cancer.

In conjunction with several types of breast cancer, our study also revealed local genetic correlations with lung cancer and lymphocytic leukemia. The locus common to SSc and lymphocytic leukemia (chr20:44072211–45673603) showed inconsistent evidence of pleiotropy, while the 6p locus (chr6:30715007–31106493), which was shared between SSc and lung cancer displayed robust evidence of gene-level pleiotropy with ten genes. These genes suggest shared mechanisms between SSc and lung cancer, pertaining to extracellular matrix (ECM) remodeling, collagen signaling and aberrant tyrosine-kinase-mediated cell adhesion.

ECM remodeling was markedly altered in both SSc and lung cancer, underscoring ECM’s role in fibrosis and tumor progression [59,60]. SSc and SSc-related interstitial lung disease featured aberrant deposition of collagen and other ECM proteins leading to progressive fibrosis of organs, including lungs [60,61]. In lung cancer, ECM with altered collagens supported tumor microenvironment by facilitating tumorigenesis, invasion and metastasis [62].

Gene enrichment for pathways related to peptidyl-tyrosine autophosphorylation and cell adhesion provided additional context for mechanistic overlap between SSc and lung cancer. Tyrosine phosphorylation was a key regulator of multiple cellular processes disrupted in SSc, namely, autoimmunity, fibrosis, and vasculopathy [63]. Dysregulation of tyrosine kinases also influences cell proliferation, adhesion, migration and metastasis in lung cancer [64].

Genes involved in epithelial function were among pleiotropic candidates associated with SSc and lung cancer. These genes maintained mucosal barriers and regulated intercellular signaling [65] and were implicated in inflammation-related oncogenic pathways [66,67]; their overexpression, has been linked to lung adenocarcinoma growth, invasion, and metastasis [65,68].

Colocalization and fine-mapping showed that the locus that was shared between SSc and lung cancer harbored distinct causal SNPs underlying these traits. These findings highlight that local genetic correlation and gene-based pleiotropy do not necessarily reflect shared causal variants, as LD and polygenicity can generate regional signals without true colocalization.

We could not successfully replicate local genetic correlations using data from FinnGen cohort. Low number of SSc cases and unique genetic background of the FinnGen cohort might have contributed to non-significant local genetic correlations and overall lower statistical power because genetic variants that are common in Europeans can be rare or absent in Finish population. Isolated populations like Finland often accumulate deleterious mutations that occur at a lower frequencies in other Europeans [69]. Sufficient differences in the genetic profiles of these populations could diminish power to detect associations and lead to failed replication attempts.

Other limitations of our study included small GWAS samples, such as in the case of hematological malignancies, which decreased overall statistical power. Additionally, GWAS on cancer included close to 10 million markers whereas the largest to date SSc GWAS covered only 4 million variants, contributing to modest marker overlap. Moreover, common susceptibility variants identified by GWAS confer incremental increases in the disease risk, explaining only small proportion of overall trait heritability. Missing heritability is attributed to rare markers and structural genetic variation [70] that can be explored in future genome-sequencing studies. It has been also shown, that local genetic correlation estimation methods, such as LAVA, generally have lower power to detect genetic correlation between binary traits compared to continuous traits [71]. In addition to potential power limitations, we analyzed traits derived from individuals of European descent potentially limiting generalizability of our findings [72,73]. Future investigations should focus on exploration of genetic correlations between SSc and cancer in non-European populations.

Importantly, different disease subsets characterized by specific autoantibody profiles have been identified in SSc [74]. We analyzed published GWAS on SSc that did not include data on specific autoantibodies; analyses stratified by autoantibody profiles and other clinical characters of patients with SSc would help better understand pathoetiological mechanisms underlying cancer development in SSc. Although, our analyses were controlled for potential sample overlap between SSc and analyzed cancer studies, there was still a possibility that cancer patients included in SSc meta-GWAS biased the results of correlation analyses. Even though we identified several loci that could be interesting candidates for future research, most of these loci carried genes with known role in cancer but not SSc. It is worth mentioning that observed local genetic correlations do not necessarily indicate true pleiotropy. In particular, misclassification or complex linkage-disequilibrium could lead to detection of spurious local pleiotropic signals [52].

One of the strengths of this study was rigorous quality control applied to the GWAS summary data. GWAS datasets included extensive information on genetic markers across genome, providing a rich resource for exploring genetic correlations. By contrast, case-case studies on cancer in SSc would require large samples which are less feasible given rarity of SSc. Our analyses also considered high heterogeneity of breast cancer, assessing genetic correlations with its’ most common molecular subtypes and revealing heterogeneous regional signals, that would have been missed otherwise. To comprehensively examine genetic relationship between SSc and cancer, we applied both global and local genetic correlation methods, gene-based cross-trait meta-analysis, colocalization and fine-mapping analyses. In contrast to LDSC, which required removal of the MHC due to its complexity and inflation of estimates, LAVA allowed us to assess genetic correlations within the MHC region, which was a significant risk factor associated with SSc [72]. Integration of multiple published GWAS datasets enhanced the ability to carry out more granular genetic correlation analyses, providing detailed insights into genetic relationships at different scales. The identification of shared genetic loci can expand our understanding of etiological basis underpinning a longstanding association between SSc and lung cancer, and inform the risk assessment and screening strategies for cancer in patients with SSc. The shared genetic loci identified in our study could be potentially utilized in earlier cancer detection or used in personalized treatment strategies. Genes residing within common loci can be explored as potential biomarkers and targeted by specific therapies, which would optimize treatment efficacy and attenuate adverse effects

## Conclusions

To conclude, we report twenty-three loci associated with SSc and numerous breast cancers subtypes as well as lung cancer and lymphocytic leukemia. These findings provide new insight into the pathological link between SSc and the commonly associated cancer types from a genetic perspective. The identified local pleiotropy between SSc and the studied cancer types needs further replications in more powered studies.

## Supporting information

S1 Fig Schematic illustration of study statistical analyses. (TIF)

S2 Fig Results of colocalization and fine-mapping analyses. Panel A displays summary table of hypothesis posterior probabilities (PP.H0–PP.H4) for colocalization (first row) and SuSiE signal pairs (subsequent rows). First two columns provide representative lead SNPs for systemic sclerosis (SSc) and lung cancer signals, respectively. The first row shows the colocalization result for the entire region, while subsequent rows show SuSiE signal comparisons. Panel B shows sensitivity analysis for the SuSiE analyses (SSc signal vs lung cancer signal). Manhattan-style plots of −log₁₀(p) for SSc (top) and lung cancer (bottom) across the locus; blue points indicate SNPs in the SuSiE credible set for the selected signals. Prior probabilities (top) and posterior probabilities (bottom) for hypotheses H0–H4 as the shared-causal prior (p₁₂) varies. The posterior curve shows H3 ≈ 1 across all prior settings, suggesting strong evidence for two distinct causal variants rather than colocalization (H4).
(TIF)

S1 Table Gene-level pleiotropic associations identified by GCPBayes analyses. Columns list cancer that was analyzed with SSc (“Trait paired with SSc “), genomic regions (“loci”) and gene within that region (“Gene”). Theta indicates overall probability of gene being associated with at least one of the traits. DS-based and HS-based columns provide evidence of pleiotropy based on credible intervals (CI) and median criteria under the Dirichlet Spike (DS) and Hierarchical Spike (HS) priors.
(DOCX)

S2 Table Gene-level associations with SSc and cancers. Columns list cancer that was analyzed with SSc (“Trait paired with SSc “), genomic regions (“loci”) and gene within that region (“Gene”). The base-10 logarithm of the Bayes Factor (log10BF), shows the strength of evidence for pleiotropy between SSc and cancer at a given locus with higher values suggesting stronger evidence. Local Bayesian False Discovery Rate (lBFDR) provides the probability that the observed association is a false positive. “Theta” provides overall probability of gene being associated with at least one of the traits, while “PPA SSc” and " PPA phenotype” list posterior probabilities with SSc and cancer, respectively.
(DOCX)

S3 Table Gene-set enrichment results. The source of gene list and pathways incorporated in the analyses are listed in the columns “Dataset” and “Pathway.” Field “Gene ratio” represents the proportion analyzed genes mapped to a particular pathways, while “Background ratio” shows the proportion of background genes associated with the same pathways. Column “Gene” includes genes associated with a particular pathway. “Fold enrichment” compares these two ratios and assesses the strength of enrichment. Columns “p-value” and “FDR p-value” list statistical significance of the enrichment and multiple testing adjusted statistical significance (false discovery rate).
(DOCX)
