# Supplementary material for: Local genetic correlations between systemic sclerosis and common cancer types
Source: PLoS One. 2026 May 27;21(5):e0350006. doi: 10.1371/journal.pone.0350006 (PMC13215533; doi:10.1371/journal.pone.0350006)
Supplement: S1 Table — Columns list cancer that was analyzed with SSc (“Trait paired with SSc “), genomic regions (“loci”) and gene within that region (“Gene”). Theta indicates overall probability of gene being associated with at least one of the traits. DS-based and HS-based columns provide evidence of pleiotropy based on credible intervals (CI) and median criteria under the Dirichlet Spike (DS) and Hierarchical Spike (HS) priors. (DOCX) [file pone.0350006.s003.docx]

| Trait paired with SSc | loci | Gene | Theta | DS-based CI Pleiotropy | DS-based Median Pleiotropy | HS-based CI Pleiotropy | HS-based Median Pleiotropy |
| --- | --- | --- | --- | --- | --- | --- | --- |
| Lung cancer | 6:30715007:31106493 | *PSORS1C2* | 1 | Yes | Yes | Yes | Yes |
|  |  | *HCG21* | 1 | Yes | Yes | No | Yes |
|  |  | *C6orf15* | 1 | Yes | Yes | Yes | Yes |
|  |  | *GTF2H4* | 1 | Yes | Yes | No | Yes |
|  |  | *LINC02570* | 1 | Yes | Yes | Yes | Yes |
|  |  | *DDR1-DT* | 1 | Yes | Yes | Yes | Yes |
|  |  | *MUC21* | 1 | Yes | Yes | Yes | Yes |
|  |  | *CDSN* | 1 | Yes | Yes | Yes | Yes |
|  |  | *MUCL3* | 1 | Yes | Yes | No | Yes |
|  |  | *VARS2* | 1 | Yes | Yes | No | Yes |
|  |  | *SFTA2* | 1 | Yes | Yes | No | Yes |
|  |  | *DDR1* | 1 | Yes | Yes | Yes | Yes |
|  |  | *XXbac-BPG118E17.11* | 1 | Yes | Yes | No | Yes |
|  |  | *PSORS1C1* | 1 | Yes | Yes | Yes | Yes |
|  |  | *HCG20* | 1 | Yes | Yes | Yes | Yes |
|  |  | *MUC22* | 1 | Yes | Yes | Yes | Yes |
| Lymphocytic leukemia | 20:44072211:45673603 | *SLC12A5* | 0.6 | No | Yes | No | Yes |
