# Supplementary material for: Local genetic correlations between systemic sclerosis and common cancer types
Source: PLoS One. 2026 May 27;21(5):e0350006. doi: 10.1371/journal.pone.0350006 (PMC13215533; doi:10.1371/journal.pone.0350006)
Supplement: S2 Table — Columns list cancer that was analyzed with SSc (“Trait paired with SSc “), genomic regions (“loci”) and gene within that region (“Gene”). The base-10 logarithm of the Bayes Factor (log10BF), shows the strength of evidence for pleiotropy between SSc and cancer at a given locus with higher values suggesting stronger evidence. Local Bayesian False Discovery Rate (lBFDR) provides the probability that the observed association is a false positive. “Theta” provides overall probability of gene being associated with at least one of the traits, while “PPA SSc” and " PPA phenotype” list posterior probabilities with SSc and cancer, respectively. (DOCX) [file pone.0350006.s004.docx]

| Trait paired with SSc | loci | Gene | log10BF | lBFDR | Theta | PPA SSc | PPA phenotype |
| --- | --- | --- | --- | --- | --- | --- | --- |
| HER2-enriched-like breast cancer | 6:106053916:107309327 | *RNU6-344P* | -2 | 1 | 0.012 | 0.019 | 0.014 |
|  |  | *RNU6-527P* | -2 | 0.9 | 0.056 | 0.082 | 0.063 |
|  |  | *RP1-134E15.3* | -1 | 0.9 | 0.034 | 0.037 | 0.09 |
|  |  | *LINC02526* | -2 | 1 | 0.009 | 0.011 | 0.01 |
|  |  | *LINC02836* | -5 | 1 | 0 | 0 | 0 |
|  |  | *RP11-404H14.1* | -4 | 1 | 0 | 0.001 | 0 |
|  |  | *RPL21P65* | -4 | 1 | 0 | 0.001 | 0 |
|  |  | *RP3-359N14.1* | -6 | 1 | 0 | 0 | 0 |
|  |  | *RP3-335E1.1* | -9 | 1 | 0 | 0 | 0 |
|  |  | *QRSL1* | 0 | 1 | 0 | 0 | 0 |
|  |  | *PRDM1* | -9 | 1 | 0 | 0 | 0 |
|  |  | *LINC02532* | 0 | 1 | 0 | 0 | 0 |
|  |  | *RTN4IP1* | 0 | 1 | 0 | 0 | 0 |
|  |  | *RP11-114N1.1* | 0 | 1 | 0 | 0 | 0 |
|  |  | *ATG5* | 180 | 8.7×10^-181 | 0 | 0 | 1 |
|  | 12:34340496:38108284 | *RP11-125N22.1* | -2 | 1 | 0.003 | 0.003 | 0.011 |
|  |  | *RP11-125N22.4* | -1 | 0.9 | 0.078 | 0.087 | 0.105 |
|  |  | *ZNF970P* | -4 | 1 | 0 | 0 | 0 |
|  |  | *DUX4L27* | -7 | 1 | 0 | 0 | 0 |
|  |  | *RP11-313F23.4* | 0 | 1 | 0 | 0 | 0 |
| Luminal A-like breast cancer | 4:49325139:53879571 | *RNU6-1252P* | -3 | 1 | 0.001 | 0.002 | 0.005 |
|  |  | *RP11-588F10.1* | -3 | 1 | 0 | 0 | 0.002 |
|  |  | *LINC02480* | -3 | 1 | 0 | 0 | 0.002 |
|  |  | *RP11-752D24.2* | -6 | 1 | 0 | 0 | 0 |
|  |  | *RP11-177B4.3* | -10 | 1 | 0 | 0 | 0 |
|  |  | *RASL11B* | -10 | 1 | 0 | 0 | 0 |
|  |  | *DANCR* | -12 | 1 | 0 | 0 | 0 |
|  |  | *SGCB* | -8 | 1 | 0 | 0 | 0 |
|  |  | *RP11-177B4.2* | -14 | 1 | 0 | 0 | 0 |
|  |  | *LRRC66* | -12 | 1 | 0 | 0 | 0 |
|  |  | *ERVMER34-1* | 0 | 1 | 0 | 0 | 0 |
|  |  | *DCUN1D4* | 26 | 3.3×10^-27 | 0 | 0 | 1 |
|  |  | *USP46* | 0 | 1 | 0 | 0 | 0 |
|  |  | *SPATA18* | -5 | 1 | 0 | 0 | 0 |
|  |  | *SCFD2* | 0 | 1 | 0 | 0 | 0 |
|  |  | *LINC01618* | 0 | 1 | 0 | 0 | 0 |
|  |  | *RP11-651C2.1* | 0 | 1 | 0 | 0 | 0 |
|  | 6:134313644:135544083 | *RP11-557H15.5* | -3 | 1 | 0.001 | 0.002 | 0.003 |
|  |  | *CTA-444M12.3* | -3 | 1 | 0 | 0 | 0.002 |
|  |  | *FAM8A6P* | -3 | 1 | 0 | 0 | 0.004 |
|  |  | *RP11-349J5.2* | -5 | 1 | 0 | 0 | 0 |
|  |  | *CTA-212D2.2* | -7 | 1 | 0 | 0 | 0 |
|  |  | *RP3-528L19.1* | -9 | 1 | 0 | 0 | 0 |
|  |  | *RP3-528L19.2* | -9 | 1 | 0 | 0 | 0 |
|  |  | *MYB* | 0 | 1 | 0 | 0 | 0 |
|  |  | *ALDH8A1* | 0 | 1 | 0 | 0 | 0 |
|  |  | *SLC2A12* | 0 | 1 | 0 | 0 | 0 |
|  |  | *CTA-444M12.4* | 0 | 1 | 0 | 0 | 0 |
|  |  | *SGK1* | 0 | 1 | 0 | 0 | 0 |
|  |  | *RP11-557H15.4* | 0 | 1 | 0 | 0 | 0 |
|  |  | *CT69* | 0 | 1 | 0 | 0 | 0 |
|  |  | *HBS1L* | 0 | 1 | 0 | 0 | 0 |
|  |  | *LINC01010* | 0 | 1 | 0 | 0 | 0 |
| Luminal B HER2-negative-like breast cancer | 4:38984839:39820061 | *RNU6-32P* | -3 | 1 | 0 | 0.002 | 0.002 |
|  |  | *RP11-539G18.1* | -3 | 1 | 0.001 | 0.002 | 0.004 |
|  |  | *RP11-360F5.3* | -4 | 1 | 0 | 0 | 0 |
|  |  | *RP11-539G18.4* | -6 | 1 | 0 | 0 | 0 |
|  |  | *RP11-472B18.3* | -4 | 1 | 0 | 0 | 0 |
|  |  | *RP11-472B18.1* | -9 | 1 | 0 | 0 | 0 |
|  |  | *RPL9* | 0 | 1 | 0 | 0 | 0 |
|  |  | *RP11-539G18.3* | 0 | 1 | 0 | 0 | 0 |
|  |  | *KLB* | 0 | 1 | 0 | 0 | 0 |
|  |  | *UGDH* | 0 | 1 | 0 | 0 | 0 |
|  |  | *RFC1* | 0 | 1 | 0 | 0 | 0 |
|  |  | *TMEM156* | 0 | 1 | 0 | 0 | 0 |
|  |  | *LIAS* | 0 | 1 | 0 | 0 | 0 |
|  |  | *UGDH-AS1* | 0 | 1 | 0 | 0 | 0 |
|  |  | *SMIM14* | 0 | 1 | 0 | 0 | 0 |
|  |  | *WDR19* | 0 | 1 | 0 | 0 | 0 |
|  |  | *UBE2K* | 0 | 1 | 0 | 0 | 0 |
|  |  | *KLHL5* | 0 | 1 | 0 | 0 | 0 |
|  |  | *RP11-360F5.1* | 0 | 1 | 0 | 0 | 0 |
|  | 17:25872111:27344401 | *AC010761.8* | -2 | 1 | 0.006 | 0.009 | 0.014 |
|  |  | *CPDP1* | -3 | 1 | 0.001 | 0.002 | 0.005 |
|  |  | *CTB-96E2.10* | -2 | 1 | 0.004 | 0.006 | 0.014 |
|  |  | *CTD-2008P7.11* | -3 | 1 | 0.001 | 0.001 | 0.008 |
|  |  | *CTD-2008P7.6* | -3 | 1 | 0.003 | 0.005 | 0.007 |
|  |  | *CTD-2350C19.2* | -2 | 0.9 | 0.029 | 0.034 | 0.047 |
|  |  | *DHRS13* | -2 | 1 | 0.005 | 0.009 | 0.008 |
|  |  | *PYY2* | -3 | 1 | 0.001 | 0.001 | 0.002 |
|  |  | *RAB34* | -1 | 0.9 | 0.066 | 0.082 | 0.073 |
|  |  | *RP1-66C13.1* | -3 | 1 | 0 | 0 | 0.001 |
|  |  | *RP11-192H23.6* | -1 | 0.9 | 0.096 | 0.114 | 0.109 |
|  |  | *RP11-19P22.8* | -3 | 1 | 0 | 0.001 | 0.002 |
|  |  | *RP11-20B24.4* | -2 | 1 | 0.005 | 0.007 | 0.009 |
|  |  | *RPL31P58* | -2 | 1 | 0.004 | 0.011 | 0.005 |
|  |  | *UNC119* | -3 | 1 | 0 | 0.001 | 0.002 |
|  |  | *AC010761.9* | -4 | 1 | 0 | 0 | 0 |
|  |  | *CTD-2008P7.3* | -4 | 1 | 0 | 0 | 0 |
|  |  | *CTD-2350C19.1* | -3 | 1 | 0.001 | 0 | 0.004 |
|  |  | *IFT20* | -3 | 1 | 0 | 0.004 | 0.001 |
|  |  | *RP11-138P22.1* | -4 | 1 | 0 | 0 | 0 |
|  |  | *RPL23A* | -2 | 1 | 0.007 | 0.009 | 0.01 |
|  |  | *SPAG5* | -3 | 1 | 0 | 0 | 0.001 |
|  |  | *AC010761.6* | -3 | 1 | 0.004 | 0.005 | 0.005 |
|  |  | *CTB-96E2.7* | -4 | 1 | 0 | 0 | 0 |
|  |  | *CTD-2008P7.1* | -4 | 1 | 0 | 0 | 0 |
|  |  | *RN7SL576P* | -4 | 1 | 0 | 0 | 0 |
|  |  | *RSKR* | -4 | 1 | 0 | 0 | 0 |
|  |  | *SEBOX* | -4 | 1 | 0 | 0 | 0 |
|  |  | *LYRM9* | -6 | 1 | 0 | 0 | 0 |
|  |  | *PROCA1* | -2 | 0.9 | 0.044 | 0.065 | 0.045 |
|  |  | *SLC13A2* | -6 | 1 | 0 | 0 | 0 |
|  |  | *SPAG5-AS1* | -4 | 1 | 0 | 0 | 0 |
|  |  | *TLCD1* | -3 | 1 | 0 | 0 | 0.003 |
|  |  | *TRAF4* | -5 | 1 | 0 | 0 | 0 |
|  |  | *AC024619.2* | -3 | 1 | 0 | 0 | 0.005 |
|  |  | *FLOT2* | -6 | 1 | 0 | 0 | 0 |
|  |  | *RP11-19P22.7* | -6 | 1 | 0 | 0 | 0 |
|  |  | *SDF2* | -5 | 1 | 0 | 0 | 0 |
|  |  | *TMEM199* | -5 | 1 | 0 | 0 | 0 |
|  |  | *PIGS* | -5 | 1 | 0 | 0 | 0 |
|  |  | *POLDIP2* | -7 | 1 | 0 | 0 | 0 |
|  |  | *TMEM97* | -6 | 1 | 0 | 0 | 0 |
|  |  | *CTB-96E2.2* | -6 | 1 | 0 | 0 | 0 |
|  |  | *RP11-20B24.5* | -6 | 1 | 0 | 0 | 0 |
|  |  | *KIAA0100* | -6 | 1 | 0 | 0 | 0 |
|  |  | *NEK8* | -4 | 1 | 0 | 0 | 0 |
|  |  | *VTN* | -6 | 1 | 0 | 0 | 0 |
|  |  | *SLC46A1* | -8 | 1 | 0 | 0 | 0 |
|  |  | *TNFAIP1* | -10 | 1 | 0 | 0 | 0 |
|  |  | *AC015688.3* | -11 | 1 | 0 | 0 | 0 |
|  |  | *ERAL1* | -2 | 0.9 | 0 | 0.083 | 0 |
|  |  | *LGALS9DP* | -8 | 1 | 0 | 0 | 0 |
|  |  | *KRT18P55* | -10 | 1 | 0 | 0 | 0 |
|  |  | *PHF12* | -7 | 1 | 0 | 0 | 0 |
|  |  | *SUPT6H* | -2 | 1 | 0.011 | 0.024 | 0.012 |
|  |  | *LGALS9* | -7 | 1 | 0 | 0 | 0 |
|  |  | *NLK* | -13 | 1 | 0 | 0 | 0 |
|  |  | *FOXN1* | -14 | 1 | 0 | 0 | 0 |
|  |  | *NOS2P1* | 0 | 1 | 0 | 0 | 0 |
|  |  | *SEZ6* | -8 | 1 | 0 | 0 | 0 |
|  |  | *CTB-96E2.3* | -16 | 1 | 0 | 0 | 0 |
|  |  | *NOS2* | 0 | 1 | 0 | 0 | 0 |
|  |  | *LINC01992* | 0 | 1 | 0 | 0 | 0 |
|  |  | *PIPOX* | -13 | 1 | 0 | 0 | 0 |
|  |  | *SARM1* | 0 | 1 | 0 | 0 | 0 |
|  |  | *KSR1* | 0 | 1 | 0 | 0 | 0 |
|  |  | *RP11-192H23.4* | 0 | 1 | 0 | 0 | 0 |
|  |  | *FAM222B* | 0 | 0.2 | 0 | 0.753 | 0 |
|  |  | *RP1-66C13.4* | 0 | 1 | 0 | 0 | 0 |
|  | 17:11778165:12676353 | *RP11-628O18.1* | -3 | 1 | 0.001 | 0.003 | 0.005 |
|  |  | *RP11-642C21.1* | -4 | 1 | 0 | 0 | 0 |
|  |  | *ARHGAP44-AS1* | -5 | 1 | 0 | 0 | 0 |
|  |  | *RP11-471L13.2* | -6 | 1 | 0 | 0 | 0 |
|  |  | *RPL21P122* | -11 | 1 | 0 | 0 | 0 |
|  |  | *ZNF18* | -15 | 1 | 0 | 0 | 0 |
|  |  | *RP11-1096G20.5* | 0 | 1 | 0 | 0 | 0 |
|  |  | *RP11-187D20.1* | 0 | 1 | 0 | 0 | 0 |
|  |  | *MYOCD-AS1* | 0 | 1 | 0 | 0 | 0 |
|  |  | *LINC00670* | 0 | 1 | 0 | 0 | 0 |
|  |  | *MAP2K4* | 0 | 1 | 0 | 0 | 0 |
|  |  | *DNAH9* | 0 | 1 | 0 | 0 | 0 |
|  |  | *MYOCD* | 0 | 1 | 0 | 0 | 0 |
|  | 22:23950733:25282436 | *AP000355.2* | -3 | 1 | 0.001 | 0.002 | 0.004 |
|  |  | *C22orf15* | -2 | 1 | 0.015 | 0.02 | 0.02 |
|  |  | *CHCHD10* | -3 | 1 | 0.002 | 0.002 | 0.005 |
|  |  | *GSTTP1* | -3 | 1 | 0.002 | 0.004 | 0.003 |
|  |  | *KB-1572G7.4* | -3 | 1 | 0.001 | 0.002 | 0.005 |
|  |  | *LL22NC03-N95F10.1* | -2 | 1 | 0.002 | 0.002 | 0.007 |
|  |  | *MIF* | -3 | 1 | 0.004 | 0.005 | 0.005 |
|  |  | *VPREB3* | -2 | 1 | 0.009 | 0.014 | 0.012 |
|  |  | *AP000354.2* | -3 | 1 | 0 | 0.003 | 0.001 |
|  |  | *CH17-476P10.1* | -3 | 1 | 0 | 0 | 0.002 |
|  |  | *DDT* | -3 | 1 | 0 | 0 | 0 |
|  |  | *KB-226F1.2* | -3 | 1 | 0.001 | 0.001 | 0.003 |
|  |  | *KB-318B8.7* | -3 | 1 | 0 | 0 | 0.005 |
|  |  | *LRRC75B* | -5 | 1 | 0 | 0 | 0 |
|  |  | *DDTL* | -4 | 1 | 0 | 0 | 0.001 |
|  |  | *AP000347.4* | -3 | 1 | 0 | 0 | 0.002 |
|  |  | *GGT1* | -6 | 1 | 0 | 0 | 0 |
|  |  | *MIF-AS1* | -6 | 1 | 0 | 0 | 0 |
|  |  | *KB-1572G7.3* | -7 | 1 | 0 | 0 | 0 |
|  |  | *DERL3* | -10 | 1 | 0 | 0 | 0 |
|  |  | *KB-1125A3.12* | -11 | 1 | 0 | 0 | 0 |
|  |  | *MMP11* | -10 | 1 | 0 | 0 | 0 |
|  |  | *UPB1* | -10 | 1 | 0 | 0 | 0 |
|  |  | *GGT5* | -10 | 1 | 0 | 0 | 0 |
|  |  | *SNRPD3* | -4 | 1 | 0 | 0 | 0 |
|  |  | *SUSD2* | -9 | 1 | 0 | 0 | 0 |
|  |  | *AP000350.6* | -15 | 1 | 0 | 0 | 0 |
|  |  | *RGL4* | -9 | 1 | 0 | 0 | 0 |
|  |  | *ZNF70* | -13 | 1 | 0 | 0 | 0 |
|  |  | *DRICH1* | -11 | 1 | 0 | 0 | 0 |
|  |  | *ADORA2A* | -8 | 1 | 0 | 0 | 0 |
|  |  | *AP000350.5* | -6 | 1 | 0 | 0 | 0 |
|  |  | *ASLP1* | -10 | 1 | 0 | 0 | 0 |
|  |  | *GUCD1* | -6 | 1 | 0 | 0 | 0 |
|  |  | *KB-1995A5.6* | -11 | 1 | 0 | 0 | 0 |
|  |  | *SLC2A11* | 0 | 1 | 0 | 0 | 0 |
|  |  | *AP000350.10* | 0 | 1 | 0 | 0 | 0 |
|  |  | *ADORA2A-AS1* | -1 | 0.6 | 0 | 0 | 0.385 |
|  |  | *PIWIL3* | 0 | 1 | 0 | 0 | 0 |
|  |  | *SPECC1L* | 0 | 1 | 0 | 0 | 0 |
|  |  | *AP000347.2* | 0 | 1 | 0 | 0 | 0 |
|  |  | *SPECC1L-ADORA2A* | 0 | 1 | 0 | 0 | 0 |
|  |  | *CABIN1* | 0 | 1 | 0 | 0 | 0 |
|  |  | *SGSM1* | 0 | 1 | 0 | 0 | 0 |
|  |  | *GUSBP11* | 0 | 1 | 0 | 0 | 0 |
|  |  | *SMARCB1* | 0 | 1 | 0 | 0 | 0 |
| Luminal B-like breast cancer | 1:8580988:9475966 | *RP4-633I8.3* | -3 | 1 | 0.001 | 0.002 | 0.005 |
|  |  | *RPL23AP19* | -3 | 1 | 0.001 | 0.002 | 0.001 |
|  |  | *RPL7P11* | -2 | 1 | 0.004 | 0.005 | 0.007 |
|  |  | *ENO1-AS1* | -3 | 1 | 0 | 0 | 0.002 |
|  |  | *LNCTAM34A* | -2 | 0.9 | 0 | 0.075 | 0 |
|  |  | *SLC2A7* | -9 | 1 | 0 | 0 | 0 |
|  |  | *ENO1* | -14 | 1 | 0 | 0 | 0 |
|  |  | *CA6* | -10 | 1 | 0 | 0 | 0 |
|  |  | *MIR34AHG* | -7 | 1 | 0 | 0 | 0 |
|  |  | *H6PD* | 5 | 8.9×10^-6 | 0 | 0 | 1 |
|  |  | *GPR157* | -15 | 1 | 0 | 0 | 0 |
|  |  | *SPSB1* | 0 | 1 | 0 | 0 | 0 |
|  |  | *SLC2A5* | -16 | 1 | 0 | 0 | 0 |
|  |  | *RERE* | 0 | 1 | 0 | 0 | 0 |
|  | 5:65606871:67096191 | *AC079467.1* | -3 | 1 | 0.001 | 0.002 | 0.003 |
|  |  | *MAST4-AS1* | -3 | 1 | 0 | 0.002 | 0.001 |
|  |  | *MAST4-IT1* | -2 | 1 | 0.009 | 0.011 | 0.014 |
|  |  | *PPIAP78* | -2 | 1 | 0.023 | 0.032 | 0.027 |
|  |  | *RP11-287J9.1* | -3 | 1 | 0.003 | 0.004 | 0.005 |
|  |  | *RP11-83M16.1* | -3 | 1 | 0.003 | 0.006 | 0.003 |
|  |  | *RP11-83M16.3* | -3 | 1 | 0.003 | 0.003 | 0.005 |
|  |  | *RP11-83M16.4* | -3 | 1 | 0.006 | 0.009 | 0.009 |
|  |  | *CTC-352D11.1* | -3 | 1 | 0 | 0 | 0.001 |
|  |  | *BCL9P1* | -6 | 1 | 0 | 0 | 0 |
|  |  | *LINC02229* | -7 | 1 | 0 | 0 | 0 |
|  |  | *CD180* | -7 | 1 | 0 | 0 | 0 |
|  |  | *RP11-357D18.1* | 0 | 1 | 0 | 0 | 0 |
|  |  | *LINC02242* | -16 | 1 | 0 | 0 | 0 |
|  |  | *RP11-83M16.6* | 0 | 1 | 0 | 0 | 0 |
|  |  | *RP11-305P14.2* | 0 | 1 | 0 | 0 | 0 |
|  |  | *RP11-305P14.1* | 0 | 1 | 0 | 0 | 0 |
|  | 7:82759284:83953009 | *AC079987.2* | -3 | 1 | 0 | 0.001 | 0.001 |
|  |  | *AC079799.2* | -7 | 1 | 0 | 0 | 0 |
|  |  | *PCLO* | -14 | 1 | 0 | 0 | 0 |
|  | 12:23923799:25058714 | *RP11-662I13.3* | -3 | 1 | 0.001 | 0.002 | 0.006 |
|  |  | *RN7SL38P* | -3 | 1 | 0.002 | 0.001 | 0.006 |
|  |  | *KNOP1P1* | -4 | 1 | 0 | 0 | 0.001 |
|  |  | *RP11-625L16.3* | -16 | 1 | 0 | 0 | 0 |
|  |  | *LINC00477* | 0 | 1 | 0 | 0 | 0 |
|  |  | *SOX5-AS1* | 0 | 1 | 0 | 0 | 0 |
|  |  | *RP11-625L16.1* | 17 | 9.3×10^-18 | 0 | 0 | 1 |
|  |  | *BCAT1* | 0 | 1 | 0 | 0 | 0 |
|  | 12:60317:1078397 | *RP5-1154L15.1* | -3 | 1 | 0.001 | 0.002 | 0.005 |
|  |  | *RP11-283I3.4* | -3 | 1 | 0 | 0 | 0 |
|  |  | *RP11-598F7.3* | -5 | 1 | 0 | 0 | 0 |
|  |  | *RP11-598F7.5* | -1 | 0.8 | 0.002 | 0.179 | 0.001 |
|  |  | *RP11-283I3.6* | -3 | 1 | 0.004 | 0.004 | 0.007 |
|  |  | *RP11-388A16.2* | -4 | 1 | 0 | 0 | 0 |
|  |  | *RP5-1154L15.2* | -5 | 1 | 0 | 0 | 0 |
|  |  | *SLC6A12* | -5 | 1 | 0 | 0 | 0 |
|  |  | *RP11-598F7.4* | -7 | 1 | 0 | 0 | 0 |
|  |  | *RP11-598F7.6* | -3 | 1 | 0 | 0.001 | 0 |
|  |  | *LINC02455* | -9 | 1 | 0 | 0 | 0 |
|  |  | *CCDC77* | -5 | 1 | 0 | 0 | 0 |
|  |  | *NINJ2-AS1* | 0 | 1 | 0 | 0 | 0 |
|  |  | *SLC6A13* | -14 | 1 | 0 | 0 | 0 |
|  |  | *IQSEC3* | 0 | 1 | 0 | 0 | 0 |
|  |  | *RAD52* | 27 | 5.6×10^-28 | 0 | 1 | 0 |
|  |  | *B4GALNT3* | 0 | 1 | 0 | 0 | 0 |
|  |  | *WNK1* | 115 | 3.1×10^-116 | 0 | 1 | 0 |
|  |  | *KDM5A* | 66 | 7.3×10^-67 | 0 | 0 | 1 |
|  |  | *NINJ2* | 0 | 1 | 0 | 0 | 0 |
|  | 14:38722115:40343754 | *RP11-14N4.1* | -2 | 1 | 0.016 | 0.019 | 0.023 |
|  |  | *RP11-407N17.2* | -3 | 1 | 0.001 | 0.003 | 0.002 |
|  |  | *RPL7AP2* | -2 | 1 | 0.009 | 0.024 | 0.01 |
|  |  | *CLEC14A* | -4 | 1 | 0 | 0 | 0 |
|  |  | *COILP1* | -4 | 1 | 0 | 0 | 0 |
|  |  | *RP11-407N17.4* | -4 | 1 | 0 | 0.001 | 0 |
|  |  | *RP11-407N17.5* | -4 | 1 | 0 | 0 | 0 |
|  |  | *RP11-506K19.2* | -3 | 1 | 0 | 0 | 0.001 |
|  |  | *YTHDF2P1* | -4 | 1 | 0 | 0 | 0 |
|  |  | *RP11-545M17.4* | -9 | 1 | 0 | 0 | 0 |
|  |  | *PNN* | -12 | 1 | 0 | 0 | 0 |
|  |  | *GEMIN2* | 0 | 1 | 0 | 0 | 0 |
|  |  | *TRAPPC6B* | 0 | 1 | 0 | 0 | 0 |
|  |  | *FBXO33* | 0 | 1 | 0 | 0 | 0 |
|  |  | *SEC23A* | 0 | 1 | 0 | 0 | 0 |
|  |  | *MIA2* | 0 | 1 | 0 | 0 | 0 |
|  |  | *RP11-96D24.1* | 0 | 1 | 0 | 0 | 0 |
|  |  | *LINC00639* | 0 | 1 | 0 | 0 | 0 |
| Lung cancer | 6:30715007:31106493 | *NAPGP2* | -2 | 1 | 0.006 | 0.009 | 0.014 |
|  |  | *RN7SKP186* | 0 | 0.3 | 0.339 | 0.743 | 0.337 |
|  |  | *RN7SL175P* | -2 | 0.9 | 0.022 | 0.021 | 0.05 |
|  |  | *PSORS1C2* | 5 | 8.5×10^-6 | 0.991 | 1 | 0.992 |
|  |  | *HCG21* | 43 | 7.6×10^-44 | 1 | 1 | 1 |
|  |  | *C6orf15* | 65 | 8.4×10^-66 | 1 | 1 | 1 |
|  |  | *GTF2H4* | 27 | 1.6×10^-28 | 1 | 1 | 1 |
|  |  | *LINC02570* | 75 | 1.9×10^-76 | 1 | 1 | 1 |
|  |  | *DDR1-DT* | 53 | 2.0×10^-54 | 1 | 1 | 1 |
|  |  | *MUC21* | 17 | 5.0×10^-18 | 1 | 1 | 1 |
|  |  | *CDSN* | 103 | 9.4×10^-104 | 1 | 1 | 1 |
|  |  | *MUCL3* | 65 | 4.9×10^-66 | 1 | 1 | 1 |
|  |  | *HCG22* | 112 | 6.9×10^-113 | 0 | 0 | 1 |
|  |  | *VARS2* | 86 | 7.3×10^-87 | 1 | 1 | 1 |
|  |  | *SFTA2* | 116 | 7.3×10^-117 | 1 | 1 | 1 |
|  |  | *DDR1* | 163 | 4.6×10^-164 | 1 | 1 | 1 |
|  |  | *XXbac-BPG118E17.11* | 118 | 2.5×10^-119 | 1 | 1 | 1 |
|  |  | *PSORS1C1* | 263 | 3.9×10^-264 | 1 | 1 | 1 |
|  |  | *HCG20* | 30 | 0 | 1 | 1 | 1 |
|  |  | *MUC22* | 305 | 3.8×10^-306 | 1 | 1 | 1 |
|  |  | *LINC00243* | 0 | 1 | 0 | 0 | 0 |
| Lymphocytic leukemia | 20:44072211:45673603 | *FTLP1* | -2 | 1 | 0.028 | 0.034 | 0.038 |
|  |  | *HNRNPA1P3* | -2 | 1 | 0.008 | 0.013 | 0.01 |
|  |  | *RNA5SP485* | -2 | 1 | 0.008 | 0.013 | 0.015 |
|  |  | *RP1-101A2.1* | -2 | 0.9 | 0.045 | 0.049 | 0.057 |
|  |  | *RP3-337O18.9* | -2 | 1 | 0.006 | 0.008 | 0.014 |
|  |  | *TP53RK-DT* | -2 | 1 | 0.007 | 0.011 | 0.01 |
|  |  | *OCSTAMP* | -1 | 0.9 | 0.072 | 0.086 | 0.074 |
|  |  | *RNU6ATAC38P* | -3 | 1 | 0 | 0.002 | 0 |
|  |  | *RP5-998H6.2* | -4 | 1 | 0 | 0 | 0 |
|  |  | *EPPIN* | -2 | 0.9 | 0.001 | 0.084 | 0.001 |
|  |  | *NEURL2* | -3 | 1 | 0 | 0 | 0.011 |
|  |  | *RPL5P2* | -2 | 1 | 0.001 | 0.045 | 0 |
|  |  | *CCNB1IP1P2* | -6 | 1 | 0 | 0 | 0 |
|  |  | *RP11-323C15.1* | -4 | 1 | 0 | 0 | 0 |
|  |  | *ZSWIM1* | -3 | 1 | 0.002 | 0.002 | 0.003 |
|  |  | *RP3-453C12.15* | -5 | 1 | 0 | 0 | 0 |
|  |  | *RPS2P7* | -4 | 1 | 0 | 0.001 | 0 |
|  |  | *SPINT3* | -4 | 1 | 0 | 0 | 0 |
|  |  | *SPINT4* | -5 | 1 | 0 | 0 | 0 |
|  |  | *TP53RK* | -5 | 1 | 0 | 0 | 0 |
|  |  | *UBE2C* | -4 | 1 | 0 | 0.001 | 0 |
|  |  | *WFDC6* | -4 | 1 | 0 | 0.001 | 0.001 |
|  |  | *RP3-461P17.9* | -3 | 1 | 0 | 0.004 | 0 |
|  |  | *WFDC10A* | -2 | 1 | 0 | 0.024 | 0 |
|  |  | *PLTP* | -6 | 1 | 0 | 0 | 0 |
|  |  | *TNNC2* | -5 | 1 | 0 | 0 | 0 |
|  |  | *CD40* | -2 | 1 | 0 | 0 | 0.024 |
|  |  | *EPPIN-WFDC6* | -3 | 1 | 0 | 0.006 | 0 |
|  |  | *MKRN7P* | -2 | 1 | 0 | 0.012 | 0 |
|  |  | *ZNF334* | -4 | 1 | 0 | 0 | 0 |
|  |  | *CTSA* | -4 | 1 | 0 | 0 | 0.001 |
|  |  | *MMP9* | -7 | 1 | 0 | 0 | 0 |
|  |  | *SLC12A5-AS1* | -7 | 1 | 0 | 0 | 0 |
|  |  | *SLC35C2* | -6 | 1 | 0 | 0 | 0 |
|  |  | *RP11-465L10.14* | 1 | 0.1 | 0.017 | 0.019 | 0.942 |
|  |  | *WFDC13* | -4 | 1 | 0 | 0.001 | 0 |
|  |  | *WFDC2* | -7 | 1 | 0 | 0 | 0 |
|  |  | *PCIF1* | -5 | 1 | 0 | 0 | 0 |
|  |  | *RP11-394O2.3* | -8 | 1 | 0 | 0 | 0 |
|  |  | *SNX21* | -6 | 1 | 0 | 0 | 0 |
|  |  | *ZNF840P* | -12 | 1 | 0 | 0 | 0 |
|  |  | *PIGT* | -2 | 0.9 | 0.033 | 0.032 | 0.053 |
|  |  | *ACOT8* | -8 | 1 | 0 | 0 | 0 |
|  |  | *SLC2A10* | 0 | 1 | 0 | 0 | 0 |
|  |  | *DNTTIP1* | -11 | 1 | 0 | 0 | 0 |
|  |  | *ELMO2* | -12 | 1 | 0 | 0 | 0 |
|  |  | *NCOA5* | 9 | 2.5×10^-10 | 0 | 0 | 1 |
|  |  | *ZNF335* | -5 | 1 | 0 | 0 | 0 |
|  |  | *WFDC10B* | 11 | 2.9×10^-12 | 0 | 1 | 0 |
|  |  | *RP5-981L23.7* | -13 | 1 | 0 | 0 | 0 |
|  |  | *ZNF663P* | -6 | 1 | 0 | 0 | 0 |
|  |  | *WFDC11* | 13 | 5.4×10^-14 | 0 | 1 | 0 |
|  |  | *SLC12A5* | 8 | 6.1×10^-9 | 0.632 | 0.636 | 1 |
|  |  | *ZSWIM3* | -7 | 1 | 0 | 0 | 0 |
|  |  | *WFDC9* | 7 | 2.0×10^-8 | 0 | 1 | 0 |
|  |  | *CDH22* | 0 | 1 | 0 | 0 | 0 |
|  |  | *WFDC3* | 0 | 1 | 0 | 0 | 0 |
|  |  | *SLC13A3* | 0 | 0.3 | 0 | 0.715 | 0 |
|  |  | *WFDC8* | 0 | 1 | 0 | 0 | 0 |
|  |  | *EYA2* | 0 | 1 | 0 | 0 | 0 |
| Triple-negative breast cancer | 4:38984839:39820061 | *RNU6-32P* | -3 | 1 | 0.002 | 0.006 | 0.002 |
|  |  | *RP11-539G18.1* | -3 | 1 | 0 | 0.002 | 0.001 |
|  |  | *RP11-360F5.3* | -4 | 1 | 0 | 0 | 0 |
|  |  | *RP11-539G18.4* | -7 | 1 | 0 | 0 | 0 |
|  |  | *RP11-472B18.3* | -6 | 1 | 0 | 0 | 0 |
|  |  | *RP11-472B18.1* | -8 | 1 | 0 | 0 | 0 |
|  |  | *RPL9* | -15 | 1 | 0 | 0 | 0 |
|  |  | *RP11-539G18.3* | 0 | 1 | 0 | 0 | 0 |
|  |  | *KLB* | 0 | 1 | 0 | 0 | 0 |
|  |  | *UGDH* | 0 | 1 | 0 | 0 | 0 |
|  |  | *RFC1* | 0 | 1 | 0 | 0 | 0 |
|  |  | *TMEM156* | 0 | 1 | 0 | 0 | 0 |
|  |  | *LIAS* | 0 | 1 | 0 | 0 | 0 |
|  |  | *UGDH-AS1* | 0 | 1 | 0 | 0 | 0 |
|  |  | *SMIM14* | 0 | 1 | 0 | 0 | 0 |
|  |  | *WDR19* | 0 | 1 | 0 | 0 | 0 |
|  |  | *UBE2K* | 0 | 1 | 0 | 0 | 0 |
|  |  | *KLHL5* | 0 | 1 | 0 | 0 | 0 |
|  |  | *RP11-360F5.1* | 0 | 1 | 0 | 0 | 0 |
|  | 7:64216136:65294277 | *RP11-328P23.2* | -3 | 1 | 0.001 | 0.003 | 0.002 |
|  |  | *RP11-460N20.3* | -1 | 0.7 | 0.079 | 0.311 | 0.082 |
|  |  | *RP11-667F9.2* | -2 | 1 | 0.011 | 0.019 | 0.015 |
|  |  | *RP11-746P2.3* | -2 | 1 | 0.005 | 0.009 | 0.009 |
|  |  | *RP11-797H7.6* | -2 | 1 | 0.008 | 0.021 | 0.01 |
|  |  | *RSL24D1P3* | -2 | 1 | 0.005 | 0.008 | 0.007 |
|  |  | *VN1R42P* | -2 | 1 | 0.014 | 0.032 | 0.016 |
|  |  | *MTDHP1* | -4 | 1 | 0 | 0 | 0 |
|  |  | *RNU6-1229P* | -2 | 0.9 | 0.006 | 0.056 | 0.007 |
|  |  | *RP13-157F18.2* | -3 | 1 | 0 | 0.003 | 0 |
|  |  | *SEPHS1P1* | -3 | 1 | 0 | 0.001 | 0 |
|  |  | *RP11-746P2.6* | -4 | 1 | 0 | 0 | 0 |
|  |  | *RP11-797H7.8* | -3 | 1 | 0 | 0.001 | 0.001 |
|  |  | *EEF1DP4* | -2 | 1 | 0 | 0.024 | 0 |
|  |  | *RP11-479O9.3* | -6 | 1 | 0 | 0 | 0 |
|  |  | *RP11-667F9.1* | -3 | 1 | 0 | 0.002 | 0 |
|  |  | *RP11-797H7.1* | -4 | 1 | 0 | 0 | 0 |
|  |  | *RP11-460N20.8* | -4 | 1 | 0 | 0 | 0 |
|  |  | *RP11-797H7.5* | -4 | 1 | 0 | 0.001 | 0 |
|  |  | *RP11-797H7.7* | -3 | 1 | 0 | 0.002 | 0 |
|  |  | *INTS4P2* | -6 | 1 | 0 | 0 | 0 |
|  |  | *RP11-460N20.4* | -2 | 1 | 0 | 0.011 | 0 |
|  |  | *CCT6P1* | -11 | 1 | 0 | 0 | 0 |
|  |  | *GTF2IP14* | 1 | 0.1 | 0 | 0.909 | 0 |
|  |  | *GTF2IP5* | 0 | 1 | 0 | 0 | 0 |
|  |  | *INTS4P1* | -12 | 1 | 0 | 0 | 0 |
|  |  | *ERV3-1* | 0 | 1 | 0 | 0 | 0 |
|  |  | *ZNF92* | 0 | 1 | 0 | 0 | 0 |
|  |  | *CCT6P3* | 15 | 3.0×10^-16 | 0 | 1 | 0 |
|  |  | *RP11-479O9.4* | 0 | 1 | 0 | 0 | 0 |
|  |  | *RP11-328P23.4* | 0 | 1 | 0 | 0 | 0 |
|  |  | *ZNF138* | 0 | 1 | 0 | 0 | 0 |
|  |  | *ZNF117* | 0 | 1 | 0 | 0 | 0 |
|  |  | *ZNF273* | -1 | 0.5 | 0.002 | 0.468 | 0.002 |
|  | 7:19131027:20122179 | *AC003986.5* | -2.594 | 1 | 0.002 | 0.004 | 0.004 |
|  |  | *AC003986.7* | -6.653 | 1 | 0 | 0 | 0 |
|  |  | *TWIST1* | -4.746 | 1 | 0 | 0 | 0 |
|  |  | *AC004543.2* | -5.924 | 1 | 0 | 0 | 0 |
|  |  | *POLR1F* | -3.23 | 1 | 0 | 0.001 | 0 |
|  |  | *TMEM196* | 0 | 1 | 0 | 0 | 0 |
|  |  | *AC005062.2* | 0 | 1 | 0 | 0 | 0 |
|  | 13:54684857:55576912 | *RPL13AP25* | -1.947 | 1 | 0.001 | 0.035 | 0.001 |
|  |  | *RP11-536C2.1* | -8.536 | 1 | 0 | 0 | 0 |
|  |  | *LINC00458* | 0 | 1 | 0 | 0 | 0 |
|  |  | *RP11-108H9.1* | 0 | 1 | 0 | 0 | 0 |
|  |  | *RP11-536C2.2* | 0 | 1 | 0 | 0 | 0 |
|  | 17:69245591:70495119 | *AC118653.2* | -2.823 | 1 | 0.001 | 0.004 | 0.002 |
|  |  | *RNU7-155P* | -2.818 | 1 | 0.001 | 0.002 | 0.003 |
|  |  | *RP11-57A1.1* | -2.845 | 1 | 0.001 | 0.004 | 0.001 |
|  |  | *SOX9* | -4.008 | 1 | 0 | 0 | 0 |
|  |  | *LINC01152* | -9.613 | 1 | 0 | 0 | 0 |
|  |  | *LINC02003* | 5.879 | 4.4×10^-7 | 0 | 0 | 1 |
|  |  | *RP11-166M16.1* | -10.799 | 1 | 0 | 0 | 0 |
|  |  | *LINC02097* | -11.309 | 1 | 0 | 0 | 0 |
|  |  | *ROCR* | -13.68 | 1 | 0 | 0 | 0 |
|  |  | *RP11-465C12.1* | 0 | 1 | 0 | 0 | 0 |
|  |  | *SOX9-AS1* | 0 | 1 | 0 | 0 | 0 |
|  |  | *RP11-84E24.4* | 0 | 1 | 0 | 0 | 0 |
|  |  | *LINC00511* | 0 | 1 | 0 | 0 | 0 |
|  | 18:41425455:42974165 | *KRT8P5* | -3.061 | 1 | 0 | 0.003 | 0.001 |
|  |  | *RNA5SP455* | -2.173 | 1 | 0.009 | 0.016 | 0.012 |
|  |  | *RP11-693M3.1* | -3.083 | 1 | 0 | 0.004 | 0 |
|  |  | *RP11-9H20.2* | -3.2 | 1 | 0 | 0.002 | 0 |
|  |  | *MLECP1* | -5.237 | 1 | 0 | 0 | 0 |
|  |  | *SETBP1-DT* | -4.27 | 1 | 0 | 0 | 0 |
|  |  | *RP11-846C15.2* | 0 | 1 | 0 | 0 | 0 |
|  |  | *CTC-782O7.3* | 0 | 1 | 0 | 0 | 0 |
|  |  | *SLC14A2* | 0 | 1 | 0 | 0 | 0 |
|  |  | *SETBP1* | 0 | 1 | 0 | 0 | 0 |
|  | 20:9279551:10279603 | *RP5-986I17.2* | -3.531 | 1 | 0 | 0.001 | 0 |
|  |  | *LAMP5-AS1* | -11.483 | 1 | 0 | 0 | 0 |
|  |  | *RP11-416N4.1* | -7.169 | 1 | 0 | 0 | 0 |
|  |  | *RP5-986I17.3* | -11.02 | 1 | 0 | 0 | 0 |
|  |  | *LAMP5* | 0 | 1 | 0 | 0 | 0 |
|  |  | *RP11-416N4.4* | -13.494 | 1 | 0 | 0 | 0 |
|  |  | *PARAL1* | 0 | 1 | 0 | 0 | 0 |
|  |  | *RP4-702M17.1* | 0 | 1 | 0 | 0 | 0 |
|  |  | *SNAP25* | 0 | 1 | 0 | 0 | 0 |
|  |  | *ANKEF1* | 0 | 1 | 0 | 0 | 0 |
|  |  | *PLCB4* | 0 | 1 | 0 | 0 | 0 |
|  |  | *SNAP25-AS1* | 0 | 1 | 0 | 0 | 0 |
