# Supplementary material for: Local genetic correlations between systemic sclerosis and common cancer types
Source: PLoS One. 2026 May 27;21(5):e0350006. doi: 10.1371/journal.pone.0350006 (PMC13215533; doi:10.1371/journal.pone.0350006)
Supplement: S3 Table — The source of gene list and pathways incorporated in the analyses are listed in the columns “Dataset” and “Pathway.” Field “Gene ratio” represents the proportion analyzed genes mapped to a particular pathways, while “Background ratio” shows the proportion of background genes associated with the same pathways. Column “Gene” includes genes associated with a particular pathway. “Fold enrichment” compares these two ratios and assesses the strength of enrichment. Columns “p-value” and “FDR p-value” list statistical significance of the enrichment and multiple testing adjusted statistical significance (false discovery rate). (DOCX) [file pone.0350006.s005.docx]

| Dataset | Pathway | Gene ratio | Background ratio | Fold enrichment | p-value | FDR p-value | Gene |
| --- | --- | --- | --- | --- | --- | --- | --- |
| GO:BP | negative regulation of cell-cell adhesion | 2/4 | 220/18860 | 42.86 | 8.00E-04 | 0.02 | *MUC21/CDSN* |
| GO:BP | negative regulation of cell adhesion | 2/4 | 326/18860 | 28.93 | 0.002 | 0.02 | *MUC21/CDSN* |
| GO:BP | extracellular matrix organization | 2/4 | 331/18860 | 28.49 | 0.002 | 0.02 | *C6orf15/DDR1* |
| GO:BP | extracellular structure organization | 2/4 | 332/18860 | 28.40 | 0.002 | 0.02 | *C6orf15/DDR1* |
| GO:BP | external encapsulating structure organization | 2/4 | 333/18860 | 28.32 | 0.002 | 0.02 | *C6orf15/DDR1* |
| GO:BP | peptidyl-tyrosine autophosphorylation | 1/4 | 10/18860 | 471.50 | 0.002 | 0.02 | *DDR1* |
| GO:BP | collagen-activated tyrosine kinase receptor signaling pathway | 1/4 | 12/18860 | 392.92 | 0.003 | 0.02 | *DDR1* |
| GO:BP | skin morphogenesis | 1/4 | 12/18860 | 392.92 | 0.003 | 0.02 | *CDSN* |
| GO:BP | keratinocyte development | 1/4 | 14/18860 | 336.79 | 0.003 | 0.02 | *CDSN* |
| GO:BP | collagen-activated signaling pathway | 1/4 | 16/18860 | 294.69 | 0.003 | 0.02 | *DDR1* |
| GO:BP | mammary gland alveolus development | 1/4 | 20/18860 | 235.75 | 0.004 | 0.02 | *DDR1* |
| GO:BP | mammary gland lobule development | 1/4 | 20/18860 | 235.75 | 0.00 | 0.02 | *DDR1* |
| GO:BP | branching involved in mammary gland duct morphogenesis | 1/4 | 25/18860 | 188.60 | 0.01 | 0.03 | *DDR1* |
| GO:BP | regulation of extracellular matrix disassembly | 1/4 | 26/18860 | 181.35 | 0.01 | 0.03 | *DDR1* |
| GO:BP | mammary gland duct morphogenesis | 1/4 | 34/18860 | 138.68 | 0.01 | 0.03 | *DDR1* |
| GO:BP | wound healing, spreading of cells | 1/4 | 41/18860 | 115.00 | 0.01 | 0.03 | *DDR1* |
| GO:BP | epiboly involved in wound healing | 1/4 | 41/18860 | 115.00 | 0.01 | 0.03 | *DDR1* |
| GO:BP | epiboly | 1/4 | 42/18860 | 112.26 | 0.01 | 0.03 | *DDR1* |
| GO:BP | lactation | 1/4 | 43/18860 | 109.65 | 0.01 | 0.03 | *DDR1* |
| GO:BP | amyloid fibril formation | 1/4 | 45/18860 | 104.78 | 0.01 | 0.03 | *CDSN* |
| GO:BP | mammary gland morphogenesis | 1/4 | 49/18860 | 96.22 | 0.01 | 0.03 | *DDR1* |
| GO:BP | morphogenesis of an epithelial sheet | 1/4 | 65/18860 | 72.54 | 0.01 | 0.04 | *DDR1* |
| GO:BP | embryo implantation | 1/4 | 71/18860 | 66.41 | 0.01 | 0.04 | *DDR1* |
| GO:BP | extracellular matrix disassembly | 1/4 | 72/18860 | 65.49 | 0.02 | 0.04 | *DDR1* |
| GO:BP | mammary gland epithelium development | 1/4 | 74/18860 | 63.72 | 0.02 | 0.04 | *DDR1* |
| GO:BP | regulation of extracellular matrix organization | 1/4 | 76/18860 | 62.04 | 0.02 | 0.04 | *DDR1* |
| GO:BP | keratinization | 1/4 | 82/18860 | 57.50 | 0.02 | 0.04 | *CDSN* |
| GO:BP | body fluid secretion | 1/4 | 95/18860 | 49.63 | 0.02 | 0.05 | *DDR1* |
| GO:BP | smooth muscle cell migration | 1/4 | 96/18860 | 49.11 | 0.02 | 0.05 | *DDR1* |
| GO:BP | muscle cell migration | 1/4 | 110/18860 | 42.86 | 0.02 | 0.05 | *DDR1* |
| GO:BP | peptidyl-tyrosine phosphorylation | 1/4 | 122/18860 | 38.65 | 0.03 | 0.05 | *DDR1* |
| GO:BP | peptidyl-tyrosine modification | 1/4 | 123/18860 | 38.33 | 0.03 | 0.05 | *DDR1* |
| GO:BP | gland morphogenesis | 1/4 | 129/18860 | 36.55 | 0.03 | 0.05 | *DDR1* |
| GO:BP | regulation of cell-matrix adhesion | 1/4 | 131/18860 | 35.99 | 0.03 | 0.05 | *DDR1* |
| GO:BP | protein autophosphorylation | 1/4 | 134/18860 | 35.19 | 0.03 | 0.05 | *DDR1* |
| GO:BP | mammary gland development | 1/4 | 142/18860 | 33.20 | 0.03 | 0.06 | *DDR1* |
| GO:BP | positive regulation of neuron projection development | 1/4 | 160/18860 | 29.47 | 0.03 | 0.06 | *DDR1* |
| GO:BP | branching morphogenesis of an epithelial tube | 1/4 | 166/18860 | 28.40 | 0.03 | 0.06 | *DDR1* |
| GO:BP | neuron projection extension | 1/4 | 174/18860 | 27.10 | 0.04 | 0.06 | *DDR1* |
| GO:BP | keratinocyte differentiation | 1/4 | 180/18860 | 26.19 | 0.04 | 0.06 | *CDSN* |
| GO:BP | female pregnancy | 1/4 | 197/18860 | 23.93 | 0.04 | 0.07 | *DDR1* |
| GO:BP | morphogenesis of a branching epithelium | 1/4 | 199/18860 | 23.69 | 0.04 | 0.07 | *DDR1* |
| GO:BP | morphogenesis of a branching structure | 1/4 | 214/18860 | 22.03 | 0.04 | 0.07 | *DDR1* |
| GO:BP | multi-organism reproductive process | 1/4 | 217/18860 | 21.73 | 0.05 | 0.07 | *DDR1* |
| GO:BP | positive regulation of phosphatidylinositol 3-kinase/protein kinase B signal transduction | 1/4 | 217/18860 | 21.73 | 0.05 | 0.07 | *DDR1* |
| GO:BP | epithelial cell development | 1/4 | 226/18860 | 20.86 | 0.05 | 0.07 | *CDSN* |
| GO:BP | multi-multicellular organism process | 1/4 | 227/18860 | 20.77 | 0.05 | 0.07 | *DDR1* |
| GO:BP | regulation of cell-substrate adhesion | 1/4 | 230/18860 | 20.50 | 0.05 | 0.07 | *DDR1* |
| GO:BP | ear development | 1/4 | 238/18860 | 19.81 | 0.05 | 0.07 | *DDR1* |
| GO:BP | developmental cell growth | 1/4 | 238/18860 | 19.81 | 0.05 | 0.07 | *DDR1* |
| GO:BP | cell-matrix adhesion | 1/4 | 246/18860 | 19.17 | 0.05 | 0.07 | *DDR1* |
| GO:BP | developmental growth involved in morphogenesis | 1/4 | 246/18860 | 19.17 | 0.05 | 0.07 | *DDR1* |
| GO:BP | epidermal cell differentiation | 1/4 | 255/18860 | 18.49 | 0.05 | 0.07 | *CDSN* |
| GO:BP | regulation of phosphatidylinositol 3-kinase/protein kinase B signal transduction | 1/4 | 311/18860 | 15.16 | 0.06 | 0.08 | *DDR1* |
| GO:BP | skin development | 1/4 | 329/18860 | 14.33 | 0.07 | 0.08 | *CDSN* |
| GO:BP | epithelial tube morphogenesis | 1/4 | 344/18860 | 13.71 | 0.07 | 0.09 | *DDR1* |
| GO:BP | phosphatidylinositol 3-kinase/protein kinase B signal transduction | 1/4 | 354/18860 | 13.32 | 0.07 | 0.09 | *DDR1* |
| GO:BP | positive regulation of cell projection organization | 1/4 | 367/18860 | 12.85 | 0.08 | 0.09 | *DDR1* |
| GO:BP | cell-substrate adhesion | 1/4 | 369/18860 | 12.78 | 0.08 | 0.09 | *DDR1* |
| GO:BP | regulation of body fluid levels | 1/4 | 389/18860 | 12.12 | 0.08 | 0.09 | *DDR1* |
| GO:BP | epidermis development | 1/4 | 402/18860 | 11.73 | 0.08 | 0.09 | *CDSN* |
| GO:BP | peptidyl-amino acid modification | 1/4 | 404/18860 | 11.67 | 0.08 | 0.09 | *DDR1* |
| GO:BP | regulation of cell growth | 1/4 | 414/18860 | 11.39 | 0.08 | 0.09 | *DDR1* |
| GO:BP | regulation of neuron projection development | 1/4 | 448/18860 | 10.52 | 0.09 | 0.1 | *DDR1* |
| GO:BP | wound healing | 1/4 | 453/18860 | 10.41 | 0.09 | 0.1 | *DDR1* |
| GO:BP | gland development | 1/4 | 454/18860 | 10.39 | 0.09 | 0.1 | *DDR1* |
| GO:BP | cellular component disassembly | 1/4 | 483/18860 | 9.76 | 0.10 | 0.1 | *DDR1* |
| GO:BP | cell growth | 1/4 | 499/18860 | 9.45 | 0.10 | 0.1 | *DDR1* |
| KEGG | Cornified envelope formation | 1/1 | 217/9521 | 43.88 | 0.02 | 0.02 | *CDSN* |
